# A modelling approach to estimate the transmissibility of SARS-CoV-2 during periods of high, low, and zero case incidence

**DOI:** 10.7554/eLife.78089

**Published:** 2023-01-20

**Authors:** Nick Golding, David J Price, Gerard Ryan, Jodie McVernon, James M McCaw, Freya M Shearer

**Affiliations:** 1 Telethon Kids Institute Nedlands Australia; 2 Curtin University Perth Australia; 3 Peter Doherty Institute for Infection and Immunity, The Royal Melbourne Hospital and The University of Melbourne Victoria Australia; 4 Melbourne School of Population and Global Health, The University of Melbourne Victoria Australia; 5 School of Ecosystem and Forest Sciences, The University of Melbourne Victoria Australia; 6 Murdoch Childrens Research Institute, The Royal Children’s Hospital Victoria Australia; 7 School of Mathematics and Statistics, The University of Melbourne Victoria Australia; Simon Fraser University Canada; McGill University Canada

**Keywords:** SARS-CoV-2, modelling, transmissibility, Viruses

## Abstract

Against a backdrop of widespread global transmission, a number of countries have successfully brought large outbreaks of COVID-19 under control and maintained near-elimination status. A key element of epidemic response is the tracking of disease transmissibility in near real-time. During major outbreaks, the effective reproduction number can be estimated from a time-series of case, hospitalisation or death counts. In low or zero incidence settings, knowing the potential for the virus to spread is a response priority. Absence of case data means that this potential cannot be estimated directly. We present a semi-mechanistic modelling framework that draws on time-series of both behavioural data and case data (when disease activity is present) to estimate the transmissibility of SARS-CoV-2 from periods of high to low – or zero – case incidence, with a coherent transition in interpretation across the changing epidemiological situations. Of note, during periods of epidemic activity, our analysis recovers the effective reproduction number, while during periods of low – or zero – case incidence, it provides an estimate of transmission risk. This enables tracking and planning of progress towards the control of large outbreaks, maintenance of virus suppression, and monitoring the risk posed by re-introduction of the virus. We demonstrate the value of our methods by reporting on their use throughout 2020 in Australia, where they have become a central component of the national COVID-19 response.

## Introduction

The first 12 months of the COVID-19 pandemic led to overwhelmed health systems and enormous social disruption across the globe. Government strategy and public responses to COVID-19 were highly variable. Prior to the global circulation of the Delta and Omicron variants, a small number of jurisdictions had achieved extended periods of elimination through 2020 and into early 2021, including Taiwan, Thailand, New Zealand and Australia (Rajatanavin et al., 2021; Summers et al., 2020; Golding et al., 2021). Meanwhile, parts of Europe and the Americas were heavily impacted by COVID-19 (The Lancet, 2020; Remuzzi and Remuzzi, 2020), with health systems overwhelmed by multiple explosive outbreaks. The Delta and Omicron variants — with their increased transmissibility — has led to epidemic activity, now likely to be sustained, in a number of previously low prevalence settings (Australian Government Department of Health, 2021; Tiberghien, 2021; New Zealand Government Ministry of Health, 2022; Mallapaty, 2022).

A key element of epidemic response is the close monitoring of the speed of disease spread, via estimation of the effective reproduction number ($R_{\mathrm{eff}}$) — the average number of new infections caused by an infected individual over their entire infectious period, in the presence of public health interventions and where no assumption of 100% susceptibility is made. Methods are well-established for near real-time estimation of this critical value and estimates are routinely assessed by decision-makers through the course of an epidemic (Gostic et al., 2020; Cori et al., 2013; Thompson et al., 2019; Abbott et al., 2020b; White and Pagano, 2008). When $R_{\mathrm{eff}}$ is above 1, the epidemic is estimated to be growing. If control measures, population immunity, or other factors can bring $R_{\mathrm{eff}}$ below 1, then the epidemic is estimated to be in decline. Accurate and timely estimation of $R_{\mathrm{eff}}$, and the timely adjustment of interventions in response to it, is critical for the sustainable and successful management of COVID-19.

However, when incident cases are driven to very low levels — as occurred in Australia following the first wave of COVID-19 from February to April 2020 — established methods for estimating $R_{\mathrm{eff}}$ are no longer informative. Yet the virus remained a threat, as evidenced by multiple instances of re-introduction and subsequent additional waves in Australia throughout 2020 and early 2021. Independent of whether local (and temporary) elimination was achieved, knowledge of SARS-CoV-2’s potential transmissibility and the risk of resurgence was a response priority.

Here, by making use of social and behavioural data, we demonstrate a novel method for estimating the ability of the virus to spread in a population, which is informative even when case incidence is very low or zero. In the absence of cases, our method estimates the ability of the virus, if it were present, to spread in a population, which we define as the ‘transmission potential’. We use the word ‘potential’ to distinguish this quantity from an estimate of actual transmission. When the virus is present, our method recovers the effective reproduction number and, additionally, the deviation between the $R_{\mathrm{eff}}$ and the transmission potential. Applying this method in real-time provides an estimate of the transmissibility of SARS-CoV-2 in periods of high, low, and even zero, case incidence, with a coherent and seamless transition in interpretation across the changing epidemiological situations.

Our innovative methods and workflows address a major challenge in epidemic situational awareness: assessing epidemic risk when case numbers are driven to low levels or (temporary) elimination is achieved, as frequently occurred in Australia through 2020–21 (Golding et al., 2021). We have routinely applied this method to all Australian states and territories and reported the outputs to peak national decision-making committees on a weekly basis since early May 2020. The concepts of transmission potential and $R_{\mathrm{eff}}$ have been incorporated into key instruments of government, including Australia’s national COVID-19 surveillance plan (Department of Health and Aged Care, 2021a). The transmission potential and $R_{\mathrm{eff}}$ are reported to the public through the Australian Government’s weekly Common Operating Picture (Department of Health and Aged Care, 2021b). While not addressed in this article, our methods have recently been updated to include consideration of variants of concern (Golding et al., 2021) and the effect of vaccination (Department of Health and Aged Care, 2021b) on reducing the ability of the virus to spread in the population.

### Model

In this section, we describe a novel method for estimating temporal trends in the transmissibility of SARS-CoV-2.

The effective reproduction number is the product of the number of contacts an infectious person makes and the per contact probability of infection (the latter of which depends on the nature and duration of contact) (Anderson and May, 1991). Both quantities are impacted by changes in behaviour, which are in turn driven by changes in policy, such as stay-at-home orders and handwashing advice, and the population’s perception and evaluation of risk, among other factors. The new techniques introduced here provide an estimate for how observed changes in rates of social contact and the per contact probability of infection translate to changes in the ability of the virus to spread.

**Figure 1.**
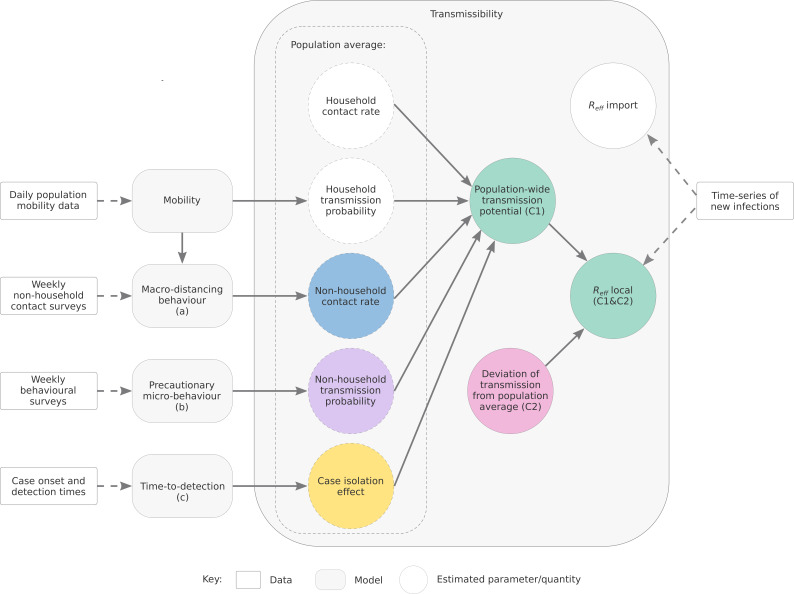
Depiction of the relationship between data sources, model components, and reported quantities.

We estimate the time-varying ability of SARS-CoV-2 to spread in a population using a novel semi-mechanistic model informed by data on cases, population behaviours and health system effectiveness (see Materials and methods). We separately model transmission from locally acquired cases (local-to-local transmission) and from overseas acquired cases (import-to-local transmission). We model local-to-local transmission ($R_{\mathrm{eff}}$) using two components (Figure 1): the average population-level trend in $R_{\mathrm{eff}}$ driven by interventions that primarily target transmission from local cases, specifically changes in physical distancing behaviour and case targeted measures (Component 1, the ‘transmission potential’ or TP); and short-term fluctuations in $R_{\mathrm{eff}}$ to capture stochastic dynamics of transmission, such as clusters of cases and short periods of lower-than-expected transmission (Component 2, the ‘deviation’ between TP and $R_{\mathrm{eff}}$). During periods of low or zero transmission, TP provides an evaluation of the ability of the virus to spread, informing risk-assessments and supporting public health planning and response (Doherty Institute, 2021).

To estimate Component 1, we use three sub-models (Figure 1, labelled a, b and c). We distinguish between two types of physical distancing behaviour:

- Macro-distancing, defined as the reduction in the average rate of non-household contacts, and assessed through weekly nationwide surveys of the daily number of non-household contacts; and
- Precautionary micro-behaviour, defined as the reduction in transmission probability per non-household contact, and assessed through weekly nationwide surveys from which we estimate the proportion of the population reporting always keeping 1.5 m physical distance from non-household contacts. Note that for Australian reporting purposes, we used the term ‘micro-distancing’ behaviour behaviour.

The modelling framework uses adherence to the 1.5 m rule as a proxy for all behaviours (other than those reducing the number of contacts) that may influence transmission, and so is intended to capture the use of masks, preference for outdoor gatherings, and hand hygiene, among other factors. The 1.5 m rule was a suitable proxy because it was consistent public health advice throughout the analysis period and time-series data were available to track adherence to this metric over time.

By synthesising data from these surveys and numerous population mobility data streams made available by technology company Google, we infer temporal trends in macro- and precautionary micro-behaviour behaviour (sub-models a and b). Furthermore, using data on the number of days from symptom onset to case notification for cases, we estimate the proportion of cases that are detected (and thus advised to isolate) by each day post-infection. By quantifying the temporal change in the probability density for the time-to-detection (sub-model c), the model estimates how earlier isolation of cases — due to improvements in contact tracing, expanded access to testing, more inclusive case definitions, and other factors impacting detection rates — reduces the ability of SARS-CoV-2 to spread.

Transmission potential (Component 1) reflects the average potential for the virus to spread at the population level. During times of disease activity, Component 2 measures how transmission within the sub-populations that have the most active cases at a given point in time differs compared to that expected from the population-wide TP. The combination of Components 1 and 2 recovers the estimated $R_{\mathrm{eff}}$ (see Equation (10) in Materials and Methods), as per established methods (Cori et al., 2013; Thompson et al., 2019; Abbott et al., 2020b). When Component 2, the deviation between TP and $R_{\mathrm{eff}}$, is positively biased ($R_{\mathrm{eff}}$ > TP), it may indicate that transmission is concentrated in populations with higher-than-average levels of mixing, such as healthcare workers or meat processing workers. If negatively biased ($R_{\mathrm{eff}}$ < TP), it reflects suppressed transmission compared to expectation. This may be due to an effective public health response actively suppressing transmission (e.g. through test, trace, isolation and quarantine), or other factors such as local depletion of susceptible individuals, and/or the virus circulating in a sub-population with fewer-than-average social contacts.

## Results

To demonstrate the utility of our method for assessing epidemic activity and risk, we report on its application to Australian data on cases, population behaviour and health system effectiveness from the first 12 months of the COVID-19 pandemic. We focus on the period from early March 2020 to late January 2021 prior to emergence of variants of concern in Australia (first Alpha, then Delta, and recently Omicron) and vaccination roll out (refer to our recent technical report for details on our approach to variants of concern Golding et al., 2021). We describe our results in the context of the COVID-19 epidemiology and public health response in Australia during this period, noting that the methods were developed and applied during the pandemic and contributed to government response efforts. We report retrospective estimates (using data as of 24 January 2021 and our model as of September 2021). Where relevant, we also report estimates made at the time of analysis in 2020, which may differ as a result of updates to the case data and methodological improvements to our model over time, as well as minor statistical variation and smoothing.

Across its eight states and territories, Australia has managed a number of distinct phases of the pandemic — from an initial wave of importations (February–April 2020), to sustained periods of zero local case incidence (April–June 2020 and October–December 2020) to widespread community transmission (June–October 2020). Like elsewhere in the world, key interventions have included quarantine of overseas arrivals, restrictions on mobility and gathering sizes, advice on personal hygiene, and case targeted interventions. The specific measures, and the level of control of SARS-CoV-2 transmission, has varied between states and over time, according to changing epidemiology and response objectives, among other factors. The model has proven informative across vastly different and rapidly changing phases of the pandemic.

To highlight these different epidemiological situations and the insights gained from our model-based analysis, we draw on exemplar events from the Australian epidemic when describing our results below. In Table 1, we summarise the key types of information provided by estimated quantities under different epidemiological situations. Further, in Figure 2—figure supplements 1–3, we provide time-series estimates of each metric and model sub-component from early March 2020 to late January 2021 for each Australian state and territory.

**Table 1.** Definitions and interpretation of key estimated quantities from the model of SARS-CoV-2 transmissibility in different epidemiological contexts. $R_{\mathrm{eff}}$ = the effective reproduction number. TP = transmission potential. C2=model component 2.

		Interpretation	
Metric	Definition	Community transmission	No transmission
TP	Expected reproduction number of a pathogen in the general population	If established in the general population, whether the epidemic is expected to grow (TP > 1) or decline (TP < 1)	Suitability for the pathogen, if it were present, to establish and maintain community transmission (TP > 1) or otherwise (TP < 1).
$R_{\mathrm{eff}}$	Average number of new infections caused by an infectious individual drawn from the active cases	Whether the epidemic is growing ($R_{\mathrm{eff}}$ > 1) or in decline ($R_{\mathrm{eff}}$ < 1)	Not applicable
C2	Deviation between TP and $R_{\mathrm{eff}}$	Whether the virus is spreading faster (C2 positively biased) or slower (C2 negatively biased) among active cases than expected	Not applicable

### Initial wave of importations

Australia took an early and precautionary approach to managing the risk of importation of SARS-CoV-2. On 1 February 2020, when China was the only country reporting uncontained transmission, Australia restricted all travel from mainland China to Australia. Only Australian citizens and residents were permitted entry from mainland China. These individuals were advised to self-quarantine for 14 days from their date of arrival. From 20 March 2020, Australia closed its borders to all foreign nationals, and from 27 March, shifted to mandatory state-managed quarantine for returned citizens and residents, with weekly quotas on the number of arrivals. These policies remained in place at the time of writing.

**Figure 2.**
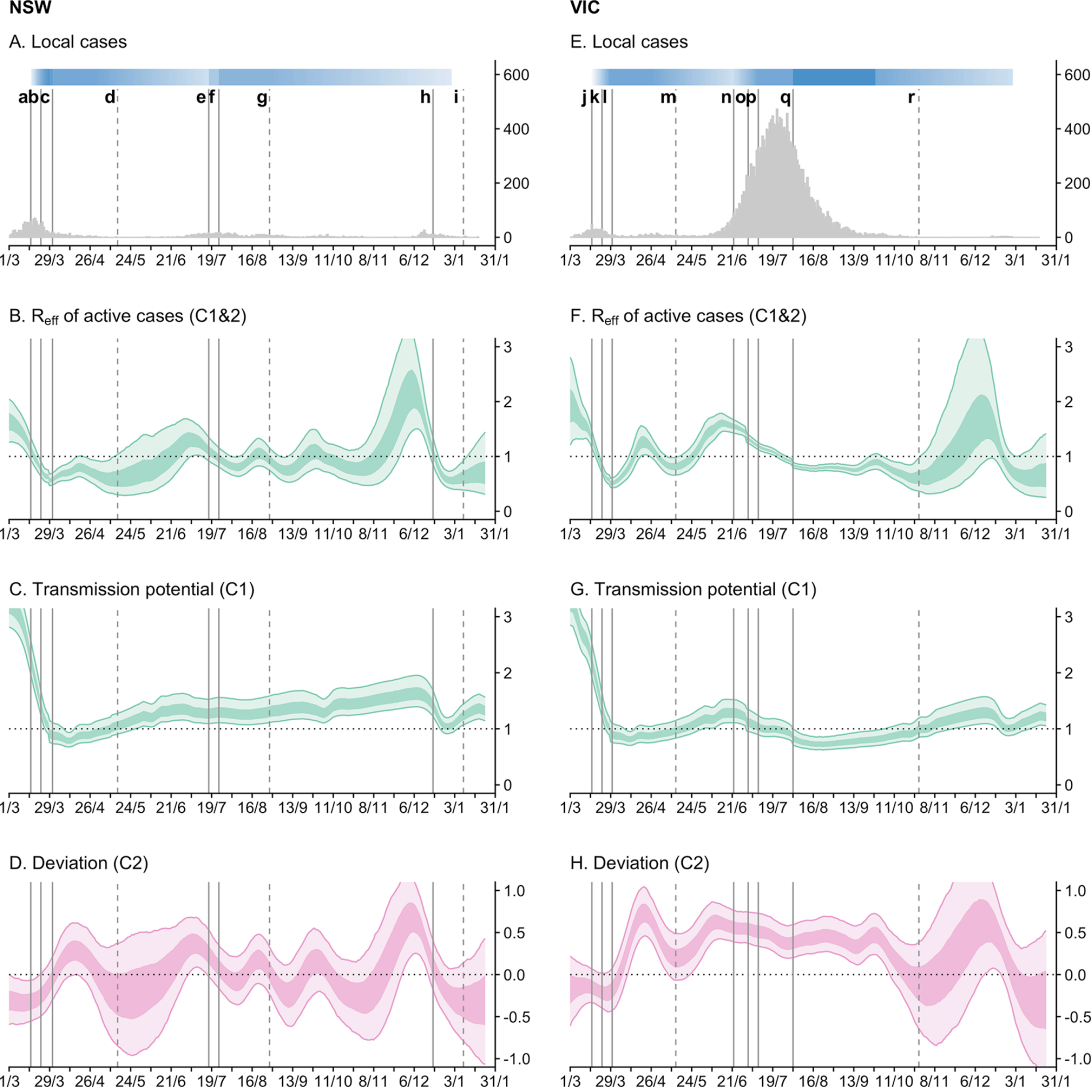
Time-series of daily local cases and transmissibility model components for the states of New South Wales (NSW) and Victoria (VIC) from 1 March 2020 to 24 January 2021. Light ribbons = 90% credible intervals; dark ribbons = 50% credible intervals. Vertical lines represent dates of key changes in restrictions on gatherings and movement, detailed in Supplementary file 1 (solid lines = tightening of restrictions; dashed lines = easing of restrictions). The blue bar is shaded according to the level of restrictions (lighter blue = less restrictions; darker blue = more restrictions). (**A and E**) Daily new local cases by inferred infection date. (**B and F**) State-wide local transmission potential (Component 1). C and G:$R_{\mathrm{eff}}$ of local active cases (Component 1&2). (**D and H**) Deviation between transmission potential and $R_{\mathrm{eff}}$ (Component 2).

During the first half of March 2020, that is, prior to the border closure, daily case incidence increased sharply. Although more than two-thirds of these cases had acquired their infection overseas, pockets of local transmission were reported in Australia’s largest cities of Sydney (New South Wales) and Melbourne (Victoria) (Australian Government Department of Health, 2020a; Figure 2A and E). From 16 March 2020, state governments progressively implemented — in rapid succession — a range of physical distancing measures to reduce and prevent community transmission. These measures were part of a coordinated national response strategy. By 31 March, Australians were strongly advised to leave their homes only for limited essential activities and public gatherings were limited to two people (known as ‘stay-at-home‘ restrictions). Health authorities also advised individuals to keep 1.5 m distance from non-household members from mid-March (Price et al., 2020).

**Figure 2—figure supplement 1.**
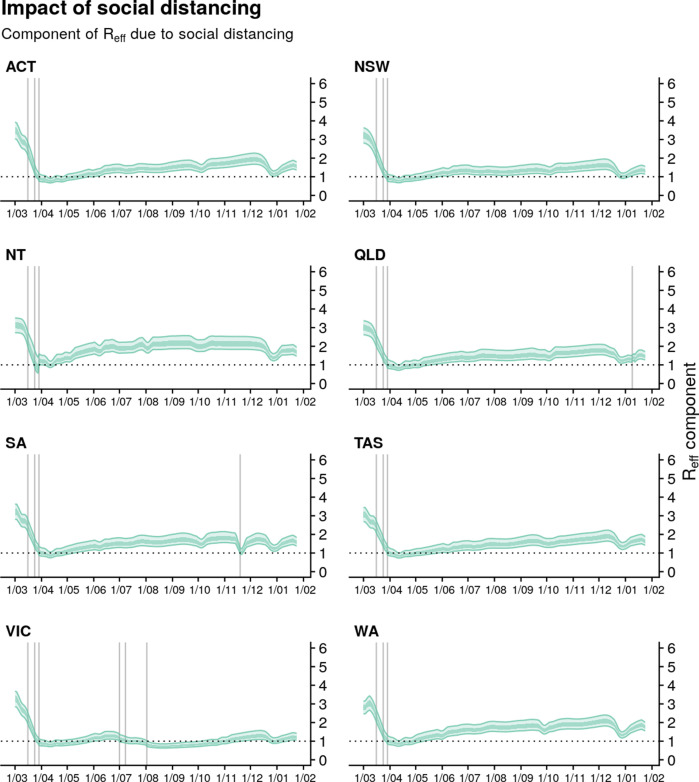
Estimates of state-wide transmission potential (Component 1) by state/territory from 1 March 2020 up to 24 January 2021 (lighter ribbons = 90% credible intervals; darker ribbons = 50% credible interval). Solid grey vertical lines indicate key dates of implementation of various physical distancing policies.

**Figure 2—figure supplement 2.**
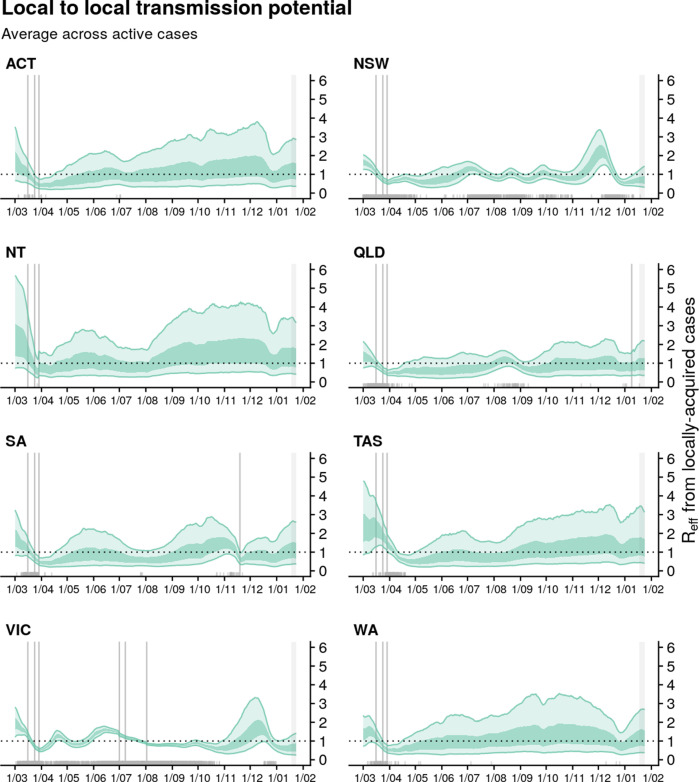
Estimates of $R_{\mathrm{eff}}$ for local active cases (model Component 1&2) for each state/territory (light green ribbon = 90% credible interval; dark green ribbon = 50% credible interval). Estimates are made from 1 March 2020 up to 24 January 2021 based on cases with inferred infection dates up to and including 18 January (due to a delay from infection to reporting, the trend in estimates after 18 January is informed by our estimates of $R_{\mathrm{eff}}$ up to 18 January and transmission potential). Solid grey vertical lines indicate key dates of implementation of various physical distancing policies. Black dotted line indicates the target value of 1 for the effective reproduction number required for control. Local cases by inferred date of infection are indicated by grey ticks on the x-axis. For states/territories with very low numbers of local active cases, the estimates of $R_{\mathrm{eff}}$ for active cases is highly uncertain. The state-wide transmission potential should be referred to when assessing the risk of an epidemic becoming established given a seeding event.

**Figure 2—figure supplement 3.**
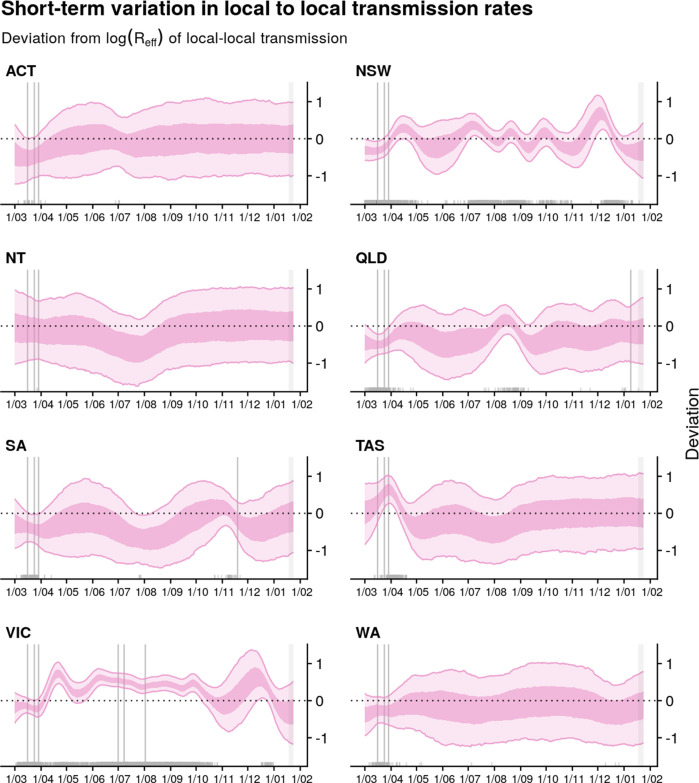
Deviation between $R_{\mathrm{eff}}$ of active cases and state-level local transmission potential (Component 2) for each state/territory (light pink ribbon = 90% credible interval; dark pink ribbon = 50% credible interval). Estimates are made from 1 March 2020 up to 24 January 2021 based on cases with inferred infection dates up to and including 18 January (due to a delay from infection to reporting, the trend in estimates after 18 January reflects the average range of deviations for that state, indicated by the grey shading). Solid grey vertical lines indicate key dates of implementation of various physical distancing policies. Local cases by inferred date of infection are indicated by grey ticks on the x-axis.

**Figure 2—figure supplement 4.**
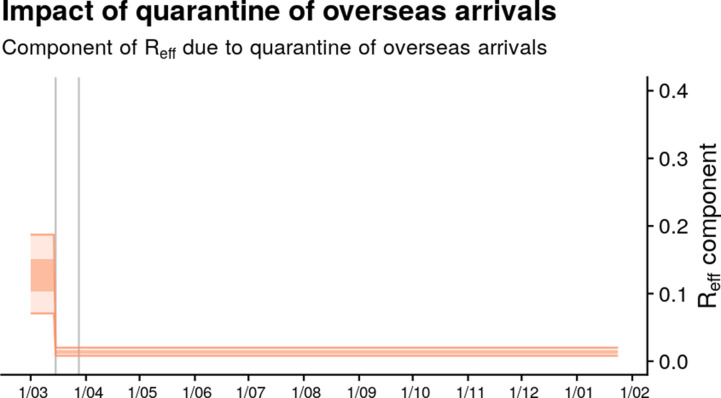
Nationwide average reduction in $R_{\mathrm{eff}}$ that is due to quarantine of overseas arrivals estimated from the $R_{\mathrm{eff}}$ model (light orange ribbon=90% credible interval; dark orange ribbon = 50% credible interval). Note that this trend does not capture time-varying fluctuations in $R_{\mathrm{eff}}$ in each state/territory. Solid grey vertical lines indicate key dates of implementation of key response policies. Note: A simple but naïve upper bound on $R_{\mathrm{eff}}$ import can be computed by assuming that all locally acquired cases arose from imported cases, and therefore computing the ratio of the numbers of local and imported cases. This results in a maximum possible value of the average $R_{\mathrm{eff}}$ import of 0.57.

**Figure 3.**
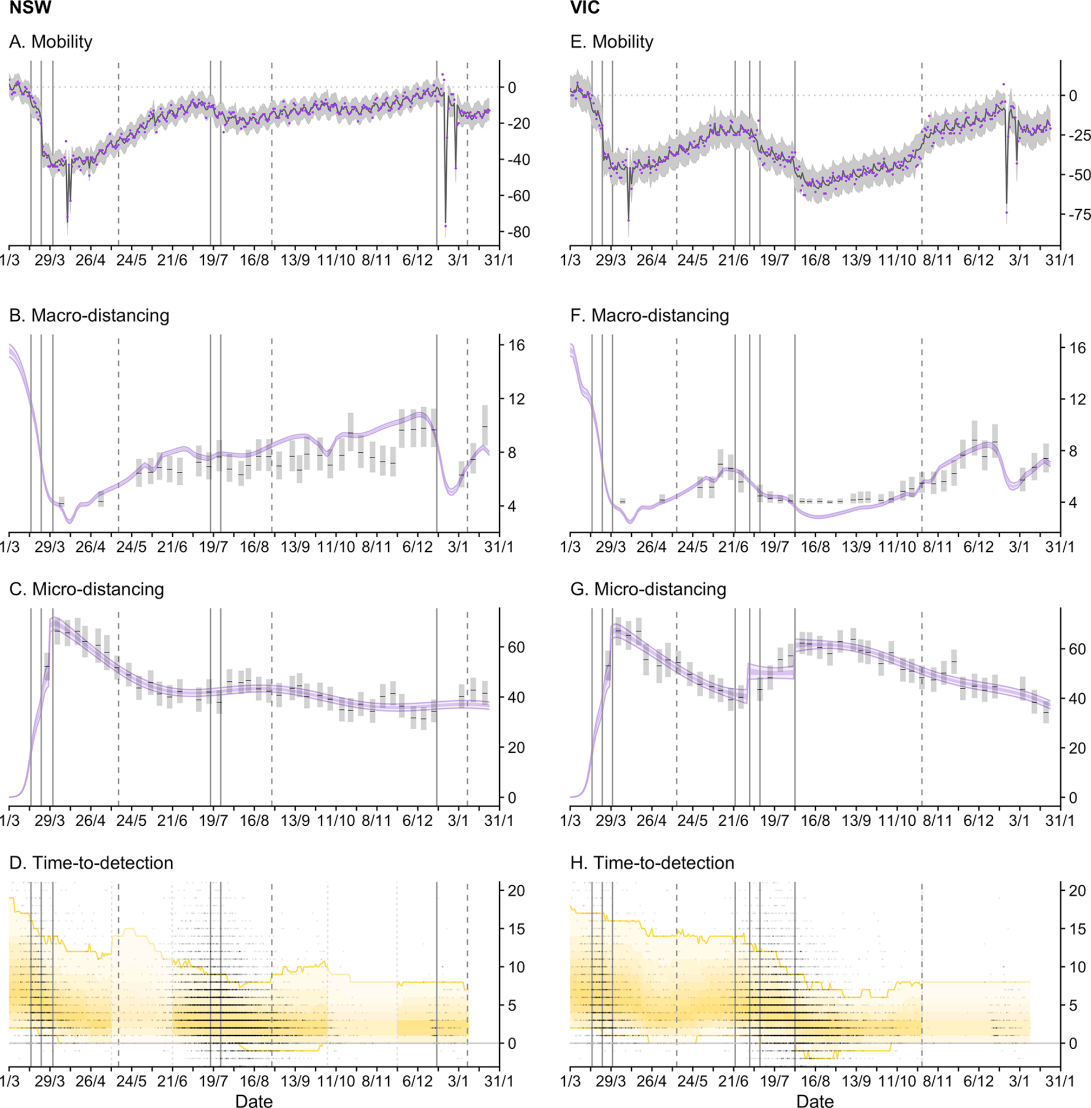
Time-series of each sub-model of transmission potential (Component 1) for New South Wales (NSW) and Victoria (VIC) from 1 March 2020 to 24 January 2021. Vertical lines represent dates of key changes in restrictions on gatherings and movement, detailed in Supplementary file 1 (solid lines = tighteningof restrictions; dashed lines = easing of restrictions). The blue bar is shaded according to the level of restrictions (lighter blue = less restrictions; darker blue = more restrictions). (**A and E**) Percentage change compared to a pre-COVID-19 baseline of one key population mobility data stream ‘Google: time a retail and recreation’. Purple dots are data stream values (percentage change on baseline). Solid lines and grey shaded regions are the estimated trend and 95% error interval estimated by our model. (**B and F**) Estimated trends in macro-distancing behaviour, that is, reduction in the daily rate of non-household contacts (dark purple ribbons = 50% credible intervals; light purple ribbons = 90% credible intervals). Estimates are informed by state-level data from nationwide weekly surveys (indicated by the black lines and grey rectangles) and population mobility data. (**C and G**) Estimated trends in precautionary micro-behaviour, that is, reduction in transmission probability per non-household contact (dark purple ribbons = 50% credible intervals, light purple ribbons = 90% credible intervals). Estimates are informed by state-level data from nationwide weekly surveys (indicated by the black lines and grey boxes). (**D and H**) Estimated trend in distributions of time from symptom onset to notification for locally acquired cases (black line = median; yellow ribbons = 90% distribution quantiles; black dots = time-to-notification of each case). Faded regions indicate where a national trend is used due to low case counts.

Through the second half of March 2020, we estimate that transmission potential across states and territories decreased substantially and rapidly from well above 1 to just below 1 (Figure 2C and G). This reflected a marked increase in macro-distancing/precautionary micro-behaviour (Figure 3B, C, F and G) and a decrease in time-to-case-detection (Figure 3D and H). Our method, with its ability to distinguish between import-to-local and local-to-local transmission, estimates that the local $R_{\mathrm{eff}}$ dropped below 1 on 22 March (upper confidence intervals) in both Victoria and New South Wales — prior to the activation of stay-at-home restrictions on 30 March (Figure 2B and F). Physical distancing measures were implemented proactively — prior to the establishment of widespread community transmission — suggesting that the effect of these measures, in combination with border measures and case-targeted interventions, led to the definitive control of a first epidemic wave.

**Figure 3—figure supplement 1.**
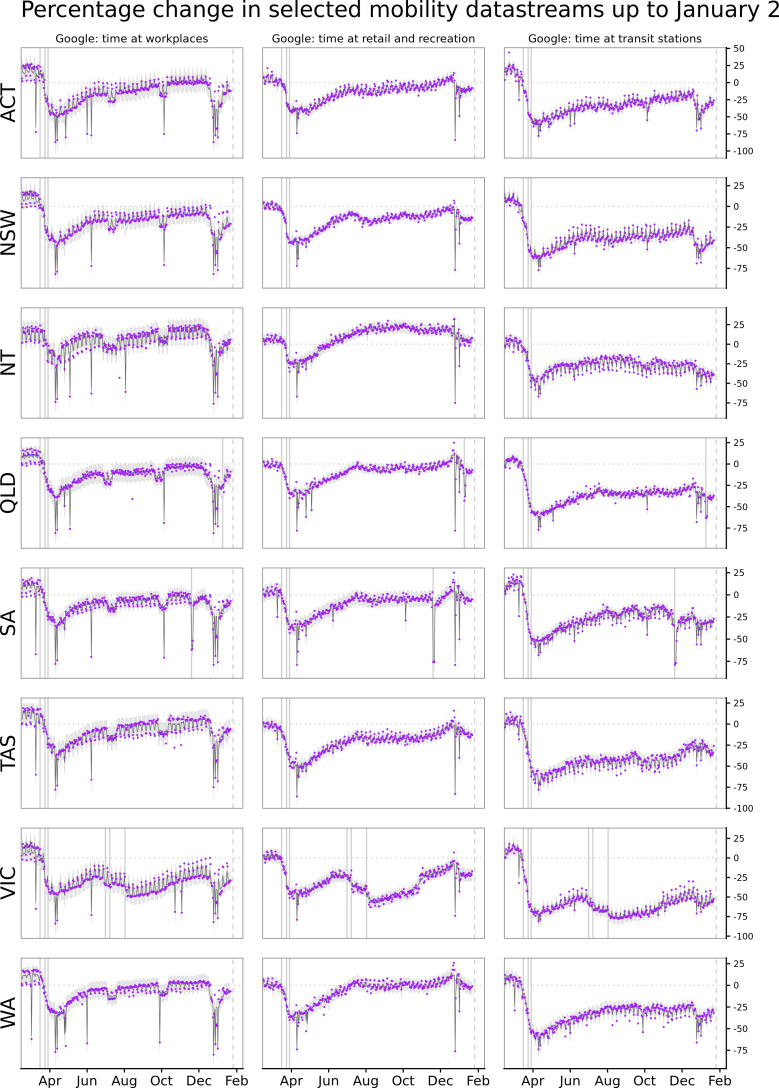
Percentage change compared to a pre-COVID-19 baseline of three key mobility data streams in each Australian state and territory from 1 March up to 24 January 2021. Solid vertical lines indicate dates of implementation of key physical distancing measures. Purple dots in each panel are data stream values (percentage change on baseline). Solid lines and grey shaded regions are the estimated trend and 95% error interval estimated by our model.

**Figure 3—figure supplement 2.**
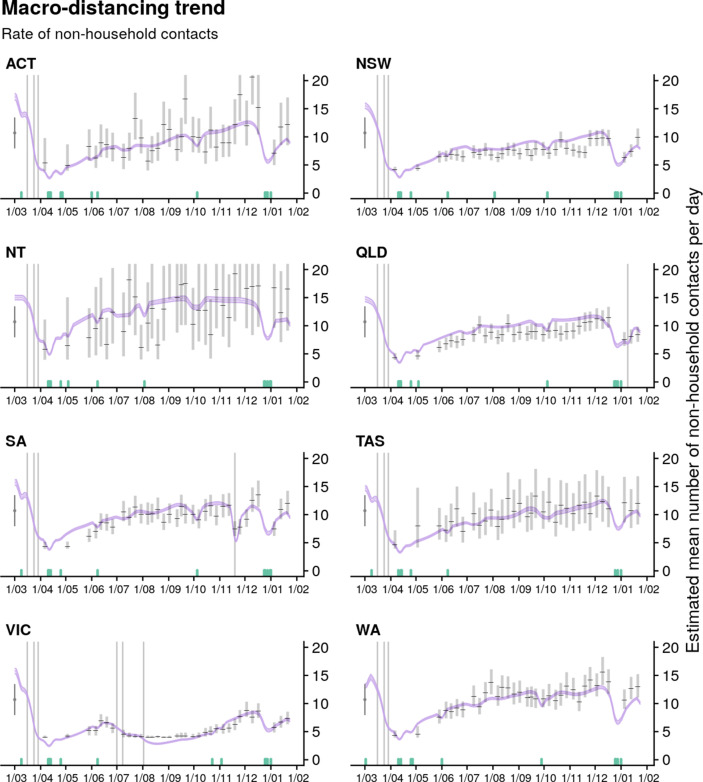
Estimated trend in macro-distancing behaviour, that is, reduction in the daily rate of non-household contacts, in each Australian state/territory from 1 March 2020 up to 24 January 2021 (light purple ribbons = 90% credible intervals; dark purple ribbons = 50% credible intervals). Estimates are informed by state-level data from nationwide surveys and population mobility data. The point wise estimates for each survey round (represented as black lines and grey rectangles) are the outputs of a separate statistical model that does not include the mobility data covariates, and is intended as a visual illustration of the level of data sparsity and variability, rather than a way of estimating fit to data, since the raw data for each week is subject to significant skew. Green ticks indicate the dates that public holidays coincided with surveys (when people tend to stay home, biasing down the number of non-household contacts reported on those days).

**Figure 3—figure supplement 3.**
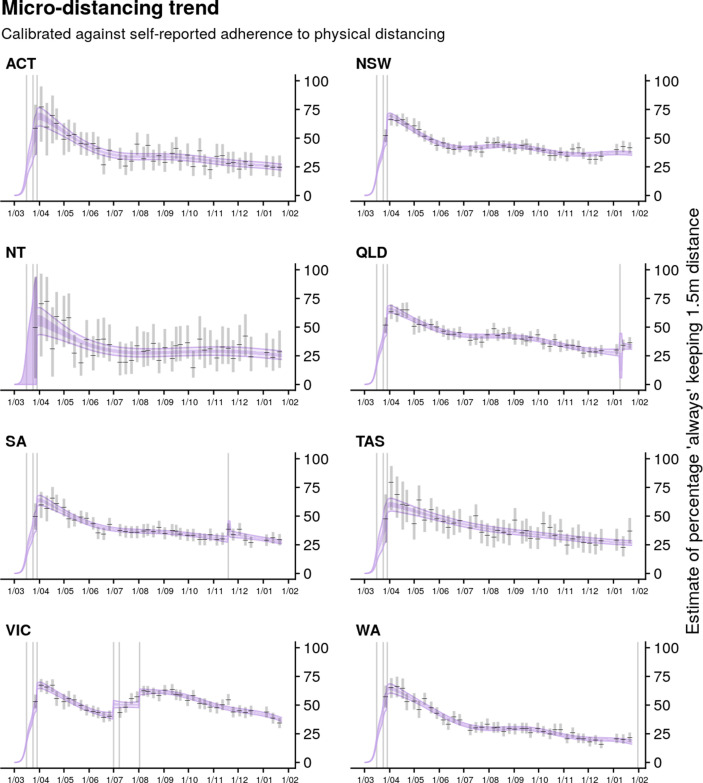
Estimated trend in precautionary micro-behaviour, that is reduction in transmission probability per non-household contact, in each Australian state/territory from 1 March 2020 up to 24 January 2021 (light purple ribbons = 90% credible intervals; dark purple ribbons = 50% credible intervals). Estimates are informed by state-level data from nationwide weekly surveys since March 2020 (indicated by the black lines and grey boxes).

**Figure 3—figure supplement 4.**
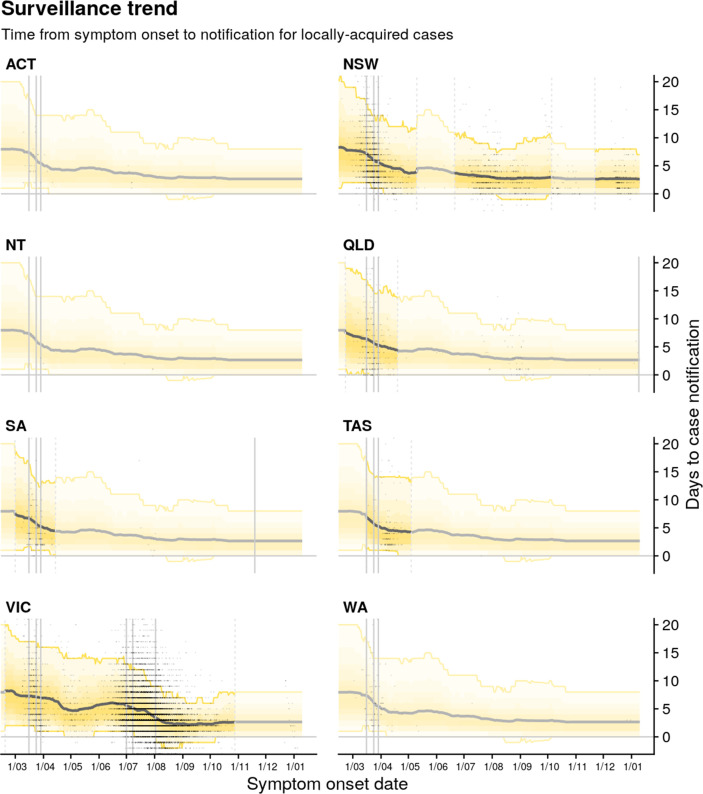
Estimated trend in distributions of time from symptom onset to notification for locally acquired cases for each Australian state/territory from 1 March 2020 to 12 January 2021 (black line = median; yellow ribbons = 90% distribution quantiles; black dots = time-to-notification of each case). Faded regions indicate where a national trend is used due to low case counts.

### Successful suppression, re-opening of society

By early April 2020, local case incidence had been driven to very low levels in all Australian states and territories. Substantial numbers of infections continued to be detected in quarantined international arrivals. However, no breaches of quarantine of significant consequence were reported until late May in the state of Victoria Lane et al., 2021.

Despite physical distancing measures remaining in place through April, levels of macro-distancing and precautionary micro-behaviour steadily waned following peak levels of adherence in the first week of April (Figure 3B, C, F, and G). This resulted in a steady increase in estimated transmission potential, although it remained below 1 suggesting that the establishment of community transmission was unlikely throughout this period (Figure 2C and G).

From May through to December 2020, the epidemiology of COVID-19 across Australia was characterised by sustained periods of zero case incidence and intermittent, localised outbreaks (with the exception of the state of Victoria, see below). With the gradual easing of restrictions from May, levels of macro-distancing and precautionary micro-behaviour continued to decrease. Accordingly, transmission potential steadily increased and by early June it had exceeded 1 in most states and territories (Figure 2), suggesting that conditions were suitable to sustain onward transmission if there were an undetected importation event or a breakdown in infection control for managed active cases/identified importations.

During the period from late June to mid-October 2020, Australia’s most populous state of New South Wales effectively controlled a series of localised outbreaks (the largest of which involved hundreds of cases). This was achieved during a period where society remained relatively open, though some restrictions on population movement and social gatherings were in place. For example, household and public gatherings were limited to 20 people. Throughout this period, as estimated at the time and now in this retrospective analysis, state-level transmission potential hovered just above 1 (Figure 2C), indicating that levels of population mixing were sufficient to allow escalation of epidemic activity in the general population in the absence of active public health measures to control outbreaks.

We estimate that $R_{\mathrm{eff}}$ oscillated around 1 throughout this period (Figure 2B). It increased to above 1 at the onset of each incursion and subsequently dropped below 1 as each cluster was contained, with no discernible change in state-level transmission potential (model Component 1) in response to each cluster. These oscillations — strong positive and then negative deviations from the transmission potential — are captured by model Component 2 and are clearly evident in the time-series (Figure 2D). Each of the positive deviations from the transmission potential are consistent with heightened transmission among clusters of cases. Each of the subsequent negative deviations from the transmission potential indicate that the number of offspring from each case of the cluster was fewer than expected given the transmission potential and estimated levels of population mixing. We interpret (and interpreted at the time) this as likely reflecting a strong public health response (i.e. early detection and isolation of cases associated with the cluster as a result of contact tracing and quarantine). This was consistent with weekly reporting on the performance of contact tracing systems in New South Wales, with 100% of cases interviewed within 24hr of notification and 100% of close contacts, identified by the case, contacted by public health officials within 48hr of case notification, from early July through to late October (Department of Health and Aged Care, 2021b).

In mid-November 2020, a sustained period of very low case incidence (i.e. zero local cases on all but 10 days in the previous 6 months) in the state of South Australia was disrupted by a breach of mandatory quarantine which led to a cluster of more than 20 cases. At the time, society was largely open with only minimal social restrictions in place. We estimate transmission potential to have been 1.71 [95% CrI: 1.47–2.01] as of 14 November in the retrospective analysis (cf. 1.27 [95% CrI: 1.14–1.41] at the time) (Figure 2), suggesting that the risk of establishing an epidemic was reasonably high (relative to the chance of stochastic extinction), and that once established, transmission would be rapid. Supported by our real-time analysis, authorities imposed a strict 3-day lockdown across the entire state to enable contact tracers to comprehensively identify and quarantine primary and secondary contacts of cases. Estimated transmission potential declined dramatically around the time of activation of restrictions, and quickly rebounded when restrictions were eased three days later (Figure 2). The incursion was rapidly contained — as result of changes to transmission potential (driven by social restrictions), an effective public health response (i.e. active case finding and management) and plausibly some favourable stochastic fluctuations — with South Australia returning to zero local case incidence from mid-December 2020.

### Resurgence of epidemic activity in one large state

In late May 2020, a breach of mandatory quarantine seeded a second epidemic wave in Australia’s second most populous state of Victoria (approximately 6.7 million people). At the time that the epidemic was seeded, many first wave restrictions were still in place. For example, gatherings within households, outdoor spaces, and dining venues were capped at 20 people, and working from home was strongly advised. Transmission potential is estimated to have been 1.07 [95% CrI: 0.88–1.22] at 25 May 2020, suggesting that levels of physical distancing may have been insufficient to prevent escalation of epidemic activity in the general population (Figure 2G).

Furthermore, from the earliest stages of the epidemic, our model estimated a strong positive deviation from the transmission potential (Component 2 positively biased, Figure 2H), corresponding to an estimate for the $R_{\mathrm{eff}}$ > 1 (95% chance of $R_{\mathrm{eff}}$ exceeding 1 by 1 June 2020 in the retrospective analysis) reflecting heightened transmission. Demographic and socio-economic assessments of the outbreak (Australian Institute of Health and Welfare, 2021; Australian Government Department of Health, 2020b; Wild et al., 2021) showed that early affected areas had higher than average household sizes and a large proportion of essential and casualised workers who were unable to work from home. Thus our model findings concurred with the observed epidemiological characteristics — that the virus was predominantly spreading in subsections of the population with higher-than-average rates of social contact — and supported public health decision making at the time.

By 1 July 2020, there were more than 600 active cases and 129 newly reported cases with an estimated $R_{\mathrm{eff}}$ of 1.33 [95% CrI: 1.25–1.41] (Figure 2F). From 9 July 2020, stay-at-home policies (denoted Stage 3 restrictions) were reinstated across metropolitan Melbourne. Despite these policies, the epidemic continued to grow through July, reaching a peak of 446 daily cases by date of symptom onset on 24 July 2020. More severe stay-at-home restrictions (denoted Stage 4) were enacted in metropolitan Melbourne on 2 August, including a night-time curfew, restrictions on movement more than 5 km from a person’s residence, and stricter definitions of essential workers and businesses including invigilation of a work permit requirement.

During the periods of Stage 3 and 4 restrictions, we observed strong increases in macro-distancing and precautionary micro-behaviour, which was reflected by a decrease in state-level transmission potential from around 1 in early June to a minimum of 0.72 [95% CrI: 0.62–0.86] on 23 August 2020 (Figure 2G), two weeks after the implementation of Stage 4 restrictions.

Following an initial sharp rise in the $R_{\mathrm{eff}}$ from well below 1 in mid-May to a peak of 1.61 [95% CrI: 1.46–1.79] at 14 June 2020, the $R_{\mathrm{eff}}$ steadily decreased over the next eight weeks (Figure 2F). We estimate that $R_{\mathrm{eff}}$ fell below the critical threshold of 1 on 25 July, approximately one week prior to the implementation of Stage 4 restrictions. With Stage 4 restrictions in place, $R_{\mathrm{eff}}$ settled between 0.8 and 1 for another eight weeks.

While both transmission potential and $R_{\mathrm{eff}}$ declined over this period, we estimated $R_{\mathrm{eff}}$ to be consistently higher than transmission potential (i.e. there was a strong positive deviation in Component 2) reflecting persistent transmission in subsections of the population with higher-than-average rates of social contact. This was consistent with other epidemiological assessments of the outbreak which suggested that transmission was concentrated in populations that were less able to physically distance (e.g. healthcare workers, residents of aged care facilities, meat workers public housing residents) (Australian Institute of Health and Welfare, 2021; Australian Government Department of Health, 2020b; Wild et al., 2021). A substantial proportion of cases were in healthcare workers and aged care facilities, particularly during the tail of the epidemic. Each of these settings required specifically targeted interventions to bring transmission under control, which were distinct from the impacts of population level measures. This may partly explain why transmission persisted for many weeks when severe stay-at-home restrictions were active, since these measures primarily target transmission in the broader community and are logically less effective at controlling transmission in essential workplaces and institutional settings.

Definitive control of the epidemic was achieved by early November 2020, when zero local case incidence reported in Victoria for the first time since April 2020.

The pattern in Component 2 for Victoria, where it deviated strongly above zero in the earliest stages of the epidemic, persisted above zero for many months, and returned to around zero once the epidemic was definitely contained, is in contrast to the oscillations seen in New South Wales from June to October.

## Discussion

We have presented a novel semi-mechanistic modelling framework for assessing transmissibility of SARS-CoV-2 from periods of high to low — or zero — case incidence, with a seamless and coherent transition in interpretation across the changing epidemiological situations. Using time-series data on cases and population behaviours, our model computes three metrics within a single framework: the effective reproduction number for active cases ($R_{\mathrm{eff}}$), the population-wide transmission potential (TP), and the deviation between $R_{\mathrm{eff}}$ and TP (C2). Our model has been applied (in real-time) to Australian data throughout the pandemic and continues to support the public health response. Here, our analysis of the first 12 months of the pandemic has demonstrated how these quantities enable the tracking and planning of progress towards the control of large outbreaks (as seen in Victoria), maintenance of virus suppression (as seen in New South Wales), and monitoring the risk posed by re-introduction of the virus (as seen in South Australia).

Our approach addresses a major challenge in epidemic situational awareness by enabling assessment of epidemic risk — via the TP — when cases are driven to low levels or (temporary) elimination is achieved. During periods of viral transmission, the model also provides new insight into epidemic dynamics via the deviation between $R_{\mathrm{eff}}$ and TP (C2). Further, the TP provides near-real-time assessment of trends in population macro-distancing and precautionary micro-behaviours that fluctuate in response to changing social restrictions, risk perception, and other factors such as school holidays. In combination, knowledge gained from $R_{\mathrm{eff}}$, TP and C2 enables policymakers to monitor the relative impacts of community-wide social restrictions and consider the need for more targeted response measures (Department of Health and Aged Care, 2021a).

Social and behavioural data have been used extensively in other countries to support COVID-19 situational assessment (Rajatanavin et al., 2021; Jarvis et al., 2020; Coletti et al., 2020; Atchison et al., 2021; Czeisler et al., 2020; Leung et al., 2021). In the UK, the CoMix study Jarvis et al., 2020 has been collecting contact data on a fortnightly basis since March 2020 and reporting “$R_{c}$" (the basic reproduction number under control measures), to the UK government’s Scientific Pandemic Influenza Group on Modelling, Operational sub-group (SPI-M-O). Conceptually, CoMix’s $R_{c}$ is akin to our TP. However, by synthesising behavioural data from multiple sources, accounting for both micro- and macro-distancing behaviours (thus estimating ‘effective’ contacts), and incorporating the effect of case surveillance, our approach is likely to capture a more complete picture of the population-wide potential for virus transmission. Further, by estimating TP and $R_{\mathrm{eff}}$ within the same modelling framework (and thus computing C2), our analysis provides a richer and more coherent epidemiological interpretation than that offered through independent measurement and reporting of each metric. Our case studies demonstrate how this richness has supported (and continues to support) the Australian COVID-19 response.

Despite its demonstrated impact, there are limitations to our approach. Firstly, it relies on data from frequent, population-wide surveys. In Australia, these data are collected for government and made available to our analysis team by a market research company which has access to an established ‘panel’ of individuals who have agreed to take part in surveys of public opinion. Researchers and governments in many other countries have used such companies for rapid data collection to support pandemic response (Jarvis et al., 2020; Atchison et al., 2021). However, these survey platforms are not readily available in all settings. Further, the sampling strategy did not allow for surveying individuals without internet access, low literacy or limited English language skills, or communication or cognitive difficulties. Further, individuals under 18 years of age were not represented in our surveys. Nor were these survey results available for the pre-pandemic period, limiting our ability to estimate what a true behavioural baseline would be for the Australian population.

The requirement for specific data streams is a limitation of our approach routinely applied in Australia in 2020 — where it was developed to address situation-specific policy questions and synthesise available data relating to the transmission process. However, the framework is modular and could be adjusted to incorporate or remove time-series of relevant quantities (e.g*.* non-household contact rates, adherence to precautionary micro-behaviour, effectiveness of surveillance), according to data availability, epidemiological relevance, and policy needs. For its use in Australia in 2020, non-household contact rates (capturing the main effects of stay-at-home measures) and precautionary micro-behaviour were considered the most important (and measurable) drivers of epidemic dynamics. In other times and places (or for other diseases), different factors may be more important for monitoring epidemic dynamics, and the variables that are quantified should be chosen accordingly.

While the patterns of TP, $R_{\mathrm{eff}}$ and C2 observed over time in Australia are consistent with “in field” epidemiological assessments, and while the methods have demonstrated impact in supporting decision making, a direct quantification of the validity of the TP is not straightforward. For example, whether self-reported adherence to the 1.5 m rule is a reliable covariate for change in the per contact probability of transmission over time is difficult to assess. If transmission were to become widespread in Australia; and therefore cases become more representative of the general population rather than specific subsets, $R_{\mathrm{eff}}$ and TP estimates would be expected to converge. However in the absence of such a natural experiment, no ground truth for this unobserved parameter exists with which to quantitatively validate the model calibration. During the Victorian second wave, while $R_{\mathrm{eff}}$ > TP is consistent with virus spread in sub-populations with higher-than-population-average rates of social contact, which was supported by other epidemiological assessments, we cannot rule out that the modelled TP was systematically underestimating the ‘true’ TP over this period.

In Australia, our methods are not only embedded in state and national situational assessment of Department of Health and Aged Care, 2021b but also national response planning. Since the model incorporates a mechanistic understanding of the impacts of physical distancing behaviour on both household and non-household transmission, it can therefore be used to predict the impact of interventions on actual and potential transmission (Doherty Institute, 2021).

Unlike other approaches that make assumptions about impacts of different interventions on behaviour, we directly measure and account for behavioural responses, providing a much more proximal way of assessing the effects of interventions (Flaxman et al., 2020). Further, while detailed data on the demographics and transmission settings for cases in Australia is unavailable, our method considers deviation (the C2) from the regional average (the TP). It is therefore less susceptible to conflation between an epidemic stochastically moving between settings of different transmissibility, and changes in population-wide transmission potential.

While not addressed in this article, our semi-mechanistic model structure enables us to perform independent estimates of the relative transmissibility of variants compared to ancestral strains. In doing so, we account for variability in the types of contacts made when low restrictions are applied (Golding et al., 2021). We are able to estimate differences between variants in the probability of transmission per unit of contact-time, for example from detailed attack rate data from overseas. These probabilities can then be combined with our estimates from Australian case data to adjust our estimates of TP under different levels of restrictions for current and emerging variants. We have also updated our modelling framework to account for the effects of vaccination on the TP (reported in the Australian Government’s Common Operating Picture from 27 August 2021 Department of Health and Aged Care, 2021b). This enables us to consider the effect of varying levels of population vaccination coverage, age-based vaccination prioritisation strategies, and levels of restrictions on the ability of the Delta variant (and future possible variants) to spread in the population. These analyses underpin the 2021 Australian national COVID-19 re-opening plan (Doherty Institute, 2021) and will be reported elsewhere. These various additions and the component models of our framework (Figure 1) provide a suite of inter-operable modules that could be used to apply the TP modelling framework to future epidemic diseases and other settings. Enabling the broader application and uptake of these methods would be aided by the development of robust research software, with the ability to modify which modules are used, to match the data streams available to the analyst. The development of such software, and detailed description of data inputs and analysis of the value of each data stream will be the focus of future work.

Our novel methods provide new insight into epidemic dynamics in both low and high incidence settings. The analyses have become an indispensable tool supporting the Australian COVID-19 response, through both situational assessment and strategic planning processes.

## Methods

### Model overview

We estimate the time-varying ability of SARS-CoV-2 to spread in a population using a novel semi-mechanistic model informed by data on cases, population behaviours and health system effectiveness. We separately model transmission from locally acquired cases (local-to-local transmission) and from overseas acquired cases (import-to-local transmission). We model local-to-local transmission ($R_{\mathrm{eff}}$) using two components:

1. The average population-level trend in transmissibility driven by interventions that primarily target transmission from local cases, specifically changes in physical distancing behaviour and case targeted measures (Component 1); and
2. Short-term fluctuations in $R_{\mathrm{eff}}$ to capture stochastic dynamics of transmission, such as clusters of cases and short periods of lower-than-expected transmission, and other factors factors influencing $R_{\mathrm{eff}}$ that are otherwise unaccounted for by the model (Component 2).

During times of disease activity, Components 1 and 2 are combined to provide an estimate of the local $R_{\mathrm{eff}}$ as traditionally measured. In the absence of disease activity, Component 1 is interpreted as the potential for the virus, if it were present, to establish and maintain community transmission (*gt*_1_) or otherwise (*lt*_1_).

### Case data

We used line-lists of reported cases for each Australian state and territory extracted from the Australian National Notifiable Diseases Surveillance System (NNDSS). The line-lists contain the date when the individual first exhibited symptoms, date when the case notification was received by the jurisdictional health department and where the infection was acquired (i.e*.* overseas or locally).

### Modelling the impact of physical distancing

#### Overview

To investigate the impact of distancing measures on SARS-CoV-2 transmission, we distinguish between two types of distancing behaviour: (1) macro-distancing that is, reduction in the rate of non-household contacts; and (2) precautionary micro-behaviour hat is, reduction in transmission probability per non-household contact.

We used data from nationwide surveys to estimate trends in specific macro-distancing (average daily number of non-household contacts) and precautionary micro-behaviour (proportion of the population always keeping 1.5 m physical distance from non-household contacts) behaviours over time. We used these survey data to infer state-level trends in macro-distancing and precautionary micro-behaviour over time, with additional information drawn from trends in mobility data.

#### Estimating changes in macro-distancing behaviour

To estimate trends in macro-distancing behaviour, we used data from: two waves of a national survey conducted in early April and early May 2020 by the University of Melbourne; and weekly waves of a national survey conducted by the Australian government from late May 2020. Respondents were asked to report the number of individuals that they had contact with outside of their household in the previous 24 hr. Note that the first wave of the University of Melbourne survey was fielded four days after Australia’s most intensive physical distancing measures were recommended nationally on 29 March 2020.

Given these data, we used a statistical model to infer a continuous trend in macro-distancing behaviour over time. This model assumed that the daily number of non-household contacts is proportional to a weighted average of time spent at different types of location, as measured by Google mobility data. The five types of places are: parks and public spaces; residential properties; retail and recreation; public transport stations; and workplaces. We fit a statistical model that infers the proportion of non-household contacts occurring in each of these types of places from:

- A survey of location-specific contact rates pre-COVID-19 Rolls et al., 2015; and
- A separate statistical model fit to the national average numbers of non-household contacts from a pre-COVID-19 contact survey and contact surveys fielded post-implementation of COVID-19 restrictions.

Waning in macro-distancing behaviour is therefore driven by Google mobility data (calibrated to survey data on non-household contact rates) on increasing time spent in each of the different types of locations since the peak of macro-distancing behaviour.

#### Estimating changes in precautionary micro-behaviour

To estimate trends in precautionary micro-behaviour, we used data from weekly national surveys (first wave from 27 to 30 March 2020) to assess changes in behaviour in response to COVID-19 public health measures. Respondents were asked to respond to the question: ‘Are you staying 1.5 m away from people who are not members of your household’ on a five point scale with response options ‘No’, ‘Rarely’, ‘Sometimes’, ‘Often’ and ‘Always’.

These behavioural survey data were used in a statistical model to infer the trend in precautionary micro-behaviour over time. Precautionary micro-behaviour was assumed to be non-existent prior to the first epidemic wave of COVID-19, and the increase in precautionary micro-behaviour to its peak was assumed to follow the same trend as precautionary micro-behaviour — implying that the population simultaneously adopted both macro-distancing and precautionary micro-behaviours around the times that restrictions were implemented.

#### Incorporating estimated changes in behaviour in the model of transmission potential

These state-level macro-distancing and precautionary micro-behaviour trends were then used in the model of transmission potential to inform the reduction in non-household transmission rates. Since the macro-distancing trend is calibrated against the number of non-household contacts, the rate of non-household transmission scales directly with this inferred trend. The probability of transmission per non-household contact is assumed to be proportional to the fraction of survey participants who report that they always maintain 1.5 m physical distance from non-household contacts. The constant of proportionality is estimated in the model of transmission potential.

The estimated rate of waning of precautionary micro-behaviour is sensitive to the metric used. If a different metric of precautionary micro-behaviour (e.g. the fraction of respondents practicing good hand hygiene) were used, this might affect the inferred rate of waning of precautionary micro-behaviour, and therefore increasing the transmission potential.

#### Modelling the impact of quarantine of overseas arrivals

We model the impact of quarantine of overseas arrivals via a ‘step function’ reflecting three different quarantine policies: self-quarantine of overseas arrivals from specific countries prior to 15 March 2020; self-quarantine of all overseas arrivals from 15 March up to 27 March 2020; and mandatory quarantine of all overseas arrivals after 27 March 2020 (Figure 2). We make no prior assumptions about the effectiveness of quarantine at reducing $R_{\mathrm{eff}}$ import, except that each successive change in policy increased that effectiveness. Note that this part of the model is intended to capture broad changes in the contribution of importation to case numbers, and is not intended to provide reliable inferences about the relative contributions of different border quarantine policies to disease importation.

#### Accounting for the impact of interstate-acquired infections

Each of Australia’s eight states and territories were modelled as a separate epidemic, with no travel assumed between jurisdictions and interstate-acquired cases handled as ‘imported cases’ within the modelling framework. We believe that these modelling decisions were reasonable for the Australian context given Australia’s unique geography (the majority of Australians live in a handful of major cities, with comparatively little movement between them), and the imposition of interstate travel restrictions during periods of COVID-19 transmission over the analysis period. Furthermore, the number of interstate importations in Australia was small and well documented in the data. Unlike overseas-acquired cases, interstate-acquired cases are assumed to contribute to onward local transmission since they were not required to quarantine.

### Model limitations

While we had access to data on whether cases are locally acquired or overseas acquired, no data were available on whether each of the locally acquired cases were infected by an imported case or by another locally acquired case. These data would allow us to disentangle the two transmission rates. Without these data, we can separate the denominators (number of infectious cases), but not the numerators (number of newly infected cases) in each group at each point in time. With access to such data, our method could provide more precise estimates of $R_{\mathrm{eff}}$.

### Model description

We developed a semi-mechanistic Bayesian statistical model to estimate $R_{\mathrm{eff}}$, or R⁢(t) hereafter, the effective rate of transmission of SARS-CoV-2 over time, whilst simultaneously quantifying the impacts on R⁢(t) of a range of policy measures introduced at national and regional levels in Australia.

#### Observation model

A straightforward observation model to relate case counts to the rate of transmission is to assume that the number of new locally acquired cases $N_{i}^{L}\,\left(t\right)$ at time t in region i is (conditional on its expectation) Poisson-distributed with mean $\lambda_{i}\,\left(t\right)$ given by the product of the total infectiousness of infected individuals $I_{i}\,\left(t\right)$ and the time-varying reproduction number $R_{i}\,\left(t\right)$:

(1) $$N_{i}^{L}\,\left(t\right)∼\mathrm{Poisson}\,\left(\lambda_{i}\,\left(t\right)\right)$$

(2) $$\lambda_{i}\,\left(t\right)=I_{i}\,\left(t\right)\,R_{i}\,\left(t\right)$$

(3) $$I_{i}\,\left(t\right)=\sum_{t^{\prime }=0}^{t}g\,\left(t^{\prime }\right)\,N_{i}\,\left(t^{\prime }\right)$$

(4) $$N_{i}\,\left(t^{\prime }\right)=N_{i}^{L}\,\left(t\right)+N_{i}^{O}\,\left(t\right)$$

where the total infectiousness, $I_{i}\,\left(t\right)$, is the sum of all active infections $N_{i}\,\left(t^{\prime }\right)$ — both locally-acquired $N_{i}^{L}\,\left(t^{\prime }\right)$ and overseas-acquired $N_{i}^{O}\,\left(t^{\prime }\right)$ — initiated at times $t^{\prime }$ prior to t, each weighted by an infectivity function $g\,\left(t^{\prime }\right)$ giving the proportion of new infections that occur $t^{\prime }$ days post-infection. The function $g\,\left(t^{\prime }\right)$ is the probability of an infector-infectee pair occurring $t^{\prime }$ days after the infector’s exposure, hat is, a discretisation of the probability distribution function corresponding to the generation interval.

This observation model forms the basis of the maximum-likelihood method proposed by White and Pagano, 2008 White and Pagano, 2008 and the variations of that method by Cori et al., 2013 Cori et al., 2013, Thompson et al., 2019 Thompson et al., 2019 and Abbott et al., 2020b Abbott et al., 2020a that have previously been used to estimate time-varying SARS-CoV-2 reproduction numbers in Australia Price et al., 2020.

We extend this model to consider separate reproduction numbers for two groups of infectious cases, in order to model the effects of different interventions targeted at each group: those with locally acquired cases $I_{i}^{L}(t)$, and those with overseas acquired cases $I_{i}^{O}\,\left(t\right)$, with corresponding reproduction numbers $R_{i}^{L}\,\left(t\right)$ and $R_{i}^{O}\,\left(t\right)$. These respectively are the rates of transmission from imported cases to locals, and from locally acquired cases to locals. We also model daily case counts as arising from a Negative Binomial distribution rather than a Poisson distribution to account for potential clustering of new infections on the same day, and use a state- and time-varying generation interval distribution $g_{i}\,\left(t^{\prime },t\right)$ (detailed in *Surveillance effect model*):

(5) $$N_{i}^{L}\,\left(t\right)∼\mathrm{NegBinomial}\,\left(\mu_{i}\,\left(t\right),r\right)$$

(6) $$\mu_{i}\,\left(t\right)=I_{i}^{L}\,\left(t\right)\,R_{i}^{L}\,\left(t\right)+I_{i}^{O}\,\left(t\right)\,R_{i}^{O}\,\left(t\right)$$

(7) $$I_{i}^{L}\,\left(t\right)=\sum_{t^{\prime }=0}^{t}g_{i}\,\left(t,t^{\prime }\right)\,N_{i}^{L}\,\left(t\right)$$

(8) $$I_{i}^{O}\left(t\right)=\sum_{t^{\prime }=0}^{t}g_{i}\left(t{,}^{\prime }t\right)N_{i}^{O}\left(t\right)$$

where the negative binomial distribution is parameterised in terms of its mean $\mu_{i}\,\left(t\right)$ and dispersion parameter r. In the commonly used probability and dispersion parameterisation with probability ψ the mean is given by μ=ψ⁢r/(1-ψ).

Note that if data were available on the whether the source of infection for each locally acquired case was another locally-acquired case or an overseas-acquired cases, we could split this into two separate analyses using the observation model above; one for each transmission source. In the absence of such data, the fractions of all transmission attributed to sources of each type is implicitly inferred by the model, with an associated increase in parameter uncertainty.

We provide the model with additional information on the rate of import-to-local transmission by adding a further likelihood term to the model for known events of import-to-local transmission since the implementation of mandatory hotel quarantine:

(9) $$K∼\mathrm{Poisson}\,\left(\sum_{i=1}^{8}\sum_{t=\tau_{2}}^{\tau_{3}}R_{i}^{O}\,\left(t\right)\,N_{i}^{0}\,\left(t\right)\right)$$

where K is the total number of known events of transmission *from* overseas-acquired cases occurring within Australia from $\tau_{2}$ = 2020-03-28 to $\tau_{3}$ = 2020-12-31. These events are largely transmission events within hotel quarantine facilities, some of which led to outbreaks of local-to-local transmission. Prior to this period, import-to-local transmission events cannot be reliably distinguished from local-to-local transmission events.

When estimating $R_{\mathrm{eff}}$ from recent case count data, care must be taken to account for under-reporting of recent cases (those which have yet to be detected), because failing to account for this under-reporting can lead to estimates of $R_{\mathrm{eff}}$ that are biased downwards. We correct for this right-truncation effect by first estimating the fraction of locally-acquired cases on each date that we would expect to have detected by the time the model is run (detection probability), and correcting both the infectiousness terms $I_{i}^{L}\,\left(t\right)$, and the observed number of new cases $N_{i}^{L}\,\left(t\right)$. We calculate the detection probability for each day in the past from the empirical cumulative distribution function of delays from assumed date of infection to date of detection over a recent period (see *Surveillance effect model*). We correct the infectiousness estimates $I_{i}^{L}\,\left(t\right)$ by dividing the number of newly infected cases on each day $N_{i}^{L}\,\left(t\right)$ by this detection probability — to obtain the expected number of new infections per day — before summing across infectiousness. We correct the observed number of new infections by a modification to the negative binomial likelihood; multiplying the expected number of cases by the detection probability to obtain the expected number of cases observed in the (uncorrected) time series of locally-acquired cases.

#### Reproduction rate models

We model the onward reproduction numbers for overseas-acquired and locally-acquired cases in a semi-mechanistic way. Reproduction numbers for local-to-local transmission are modelled as a combination of a deterministic model of the population-wide transmission potential for that type of case, and a correlated time series of random effects to represent stochastic fluctuations in the reporting rate in each state over time. Import-to-local transmission is modelled in a mechanistic way:

(10) $$R_{i}^{L}\,\left(t\right)=\exp\left(\log\left(R_{i}^{*}\,\left(t\right)\right)-\sigma^{2}/2+\epsilon_{i}\,\left(t\right)\right)$$

(11) $$R_{i}^{O}\,\left(t\right)=R_{i}^{*}\,\left(0\right)\,Q\,\left(t\right)$$

For both locally acquired and overseas-acquired infections, the effective reproduction number depends on the transmission potential $R_{i}^{*}(t)$ is given by a deterministic epidemiological model of population-wide transmission potential that considers the effects of distancing behaviours. The correlated time series of random effects $\epsilon_{i}\,\left(t\right)$ represents stochastic fluctuations in these local-local reproduction numbers in each state over time — for example due to clusters of transmission in sub-populations with higher or lower reproduction numbers than the general population. We consider that the transmission potential $R_{i}^{*}\,\left(t\right)$ is the average of individual reproduction numbers over the entire state population, whereas the effective reproduction number $R_{i}^{L}\,\left(t\right)$ is the average of individual reproduction numbers among a (non-random) sample of individuals – those that make up the active cases at that point in time. We therefore expect that the long-term average of $R_{i}^{L}\,\left(t\right)$ will equate to $R_{i}^{*}\,\left(t\right)$. The relationship between these two is therefore defined such that the hierarchical distribution over $R_{i}^{L}\,\left(t\right)$ is marginally (with respect to time) a log-normal distribution with mean $R_{i}^{*}\,\left(t\right)$. The parameter $\sigma^{2}$ is the marginal variance of the $\epsilon_{i}$, as defined in the kernel function of the Gaussian process.

Note that in this model the random effects term $\epsilon_{i}$ and its variance term $\sigma^{2}$ is intended to have a mechanistic interpretation as the stochasticity due to random sampling (of people currently infected from the total population). It is not incorporated to account for error in specification of the transmission potential in the way that temporal random effects are commonly used in statistical modelling. Consequently, small variance in the timeseries plots of $\epsilon_{i}$ is not indicative of good fit, but of a large number of infections; as the size of the sample increases, the variance of mean decreases.

For overseas-acquired cases the population-wide transmission rate at time t, $R_{i}^{*}\,\left(0\right)\,Q\,\left(t\right)$, is the baseline rate of transmission ($R_{i}^{*}\,\left(0\right)=R_{0}$; local-to-local transmission potential in the absence of distancing behaviour or other mitigation) multiplied by a quarantine effect model, Q⁢(t), that encodes the efficacy of the three different overseas quarantine policies implemented in Australia (described below).

We model $R_{i}^{*}\,\left(t\right)$, the population-wide rate of local-to-local transmission at time t, as the sum of two components: the rate of transmission to members of the same household, and to members of other households. Each of these components is computed as the product of the number of contacts, and the probability of transmission per contact. The transmission probability is in turn modelled as a binomial process considering the duration of contact with each person and the probability of transmission per unit time of contact. This mechanistic consideration of the contact process enables us to separately quantify how macro-distancing and precautionary micro-behaviours impact on transmission, and to make use of various ancillary measures of both forms of distancing:

(12) $$R_{i}^{*}\,\left(t\right)=s_{i}\,\left(t\right)\,\left(H\,C_{0}\,\left(1-{\left(1-p\right)}^{H\,D_{0}\,h_{i}\,\left(t\right)\,d}\right)+N\,C_{0}\,\delta_{i}\,\left(t\right)\,d\,\left(1-{\left(1-p\right)}^{N\,D_{0}}\right)\,\gamma_{i}\,\left(t\right)\right)$$

where: s⁢(t) is the effect of surveillance on transmission, due to the detection and isolation of cases (detailed below); $H\,C_{0}$ and $N\,C_{0}$ are the baseline (i.e. before adoption of distancing behaviours) daily rates of contact with, respectively, people who are, and are not, members of the same household; $H\,D_{0}$ and $N\,D_{0}$ are the baseline average total daily duration of contacts with household and non-household members (measured in hours); d is the average duration of infectiousness in days; p is the probability of transmitting the disease per hour of contact, and; $h_{i}\,\left(t\right)$, $\delta_{i}\,\left(t\right)$, $\gamma_{i}\,\left(t\right)$ are time-varying indices of change relative to baseline of the duration of household contacts, the number of non-household contacts, and the transmission probability per non-household contact, respectively (modifying both the duration and transmission probability per unit time for non-household contacts).

The first component in Equation (12) is the rate of household transmission, and the second is the rate of non-household transmission. Note that the duration of infectiousness d is considered differently in each of these components. For household members, the daily number of household contacts is typically close to the total number of household members, hence the expected number of household transmissions asymptotically approaches the household size; so the number of days of infectiousness contributes to the probability of transmission to each of those household members. This is unlikely to be the case for non-household members, where each day’s non-household contacts may overlap, but are unlikely to be from a small finite pool. This assumption would be unnecessary if contact data were collected on a similar timescale to the duration of infectiousness, though issues with participant recall in contact surveys mean that such data are unavailable. Note that this model does not have a household network structure, nor account for depletion of susceptible individuals within a household.

The parameters $H\,C_{0}$, $H\,D_{0}$, and $N\,D_{0}$ are all estimated from a contact survey conducted in Melbourne in 2015 Rolls et al., 2015. $N\,C_{0}$ is computed from an estimate of the total number of contacts per day for adults from Prem et al., 2017, minus the estimated rate of household contacts. Whilst Rolls et al., 2015 also provides an estimate of the rate of non-household contacts, the method of data collection (a combination of ‘individual’ and ‘group’ contacts) makes it less comparable with contemporary survey data than the estimate of Prem et al., 2017.

The expected duration of infectiousness d is computed as the mean of the non-time-varying discrete generation interval distribution:

(13) $$d=\sum_{t^{\prime }=0}^{\infty }t^{\prime }\,g*\left(t^{\prime }\right)$$

and change in the duration of household contacts over time $h_{i}\,\left(t\right)$ is assumed to be equivalent to change in time spent in residential locations in region i, as estimated by the mobility model for the data stream *Google: time at residential*. In other words, the total duration of time in contact with household members is assumed to be directly proportional to the amount of time spent at home. Unlike the effect on non-household transmission, an increase in macro-distancing is expected to slightly increase household transmission due to this increased contact duration.

The time-varying parameters $\delta_{i}\,\left(t\right)$ and $\gamma_{i}\,\left(t\right)$ respectively represent macro-distancing and precautionary micro-behaviour; behavioural changes that reduce mixing with non-household members, and the probability of transmission for each of non-household member contact. We model each of these components, informed by population mobility estimates from the mobility model and calibrated against data from nationwide surveys of contact behaviour. *Surveillance effect model* Disease surveillance — both screening of people with COVID-like symptoms and performing contact tracing — can improve COVID-19 control by placing cases in isolation so that they are less likely to transmit the pathogen to other people. Improvements in disease surveillance can therefore lead to a reduction in transmission potential by isolating cases more quickly, and reducing the time they are infectious but not isolated. Such an improvement changes two quantities: the population average transmission potential $R^{*}\,\left(t\right)$ is reduced by a factor $s_{i}\,\left(t\right)$; and the generation interval distribution $g\,\left(t,t^{\prime }\right)$ is shortened, as any transmission events are more likely to occur prior to isolation.

We model both of these functions using a region- and time-varying estimate of the survival function (one minus the cumulative density function) $f_{i}\,\left(t,t^{\prime }\right)$ of the discrete probability distribution over times from infection to detection:

(14) $$g_{i}\,\left(t,t^{\prime }\right)=\frac{f_{i}\,\left(t,t^{\prime }\right)\,g^{*}\,\left(t^{\prime }\right)}{s_{i}\,\left(t\right)}$$

(15) $$s_{i}\,\left(t\right)=\sum_{t^{\prime }=0}^{\infty }f_{i}\,\left(t,t^{\prime }\right)\,g^{*}\,\left(t^{\prime }\right)$$

where $g^{*}\,\left(t^{\prime }\right)$ is the baseline generation interval distribution, representing times to infection in the absence of detection and isolation of cases, $s_{i}\,\left(t\right)$ is a normalising factor — and also the effect of surveillance on transmission — and $f_{i}\,\left(t,t^{\prime }\right)$ is a region- and time-varying probability density over periods from infection to isolation $t^{\prime }$. In states/territories and at times when cases are rapidly found and placed in isolation, the distribution encoded by $f_{i}\,\left(t,t^{\prime }\right)$ has most of its mass on small delays, average generation intervals are shortened, and the surveillance effect $s_{i}\,\left(t\right)$ tends toward 0 (a reduction in transmission). At times when cases are not found and isolated until after most of their infectious period has passed, $f_{i}\,\left(t,t^{\prime }\right)$ has most of its mass on large delays, generation intervals are longer on average, and $s_{i}\,\left(t\right)$ tends toward 1 (no effect of reduced transmission).

We model the region- and time-varying distributions $f_{i}\,\left(t,t^{\prime }\right)$ empirically via a time-series of empirical distribution functions computed from all observed infection-to-isolation periods observed within an adaptive moving window around each time t. Since dates of infection and isolation are not routinely recorded in the dataset analysed, we use 5 days prior to the date of symptom onset to be the assumed date of infection, and the date of case notification to be the assumed date of isolation. This will overestimate the time to isolation and therefore underestimate the effect of surveillance when a significant proportion of cases are placed into isolation prior to testing positive — for example, during the tail of an outbreak being successfully controlled by contact tracing.

For a given date and state/territory, the empirical distribution of delays from symptom onset to notification is computed from cases with symptom onset falling within a time window around that date, with the window selected to be the smallest that will yield at least 500 observations; but constrained to between one and eight weeks.

Where a state/territory does not have sufficient cases to reliably estimate this distribution in an eight week period, a national estimate is used instead. Specifically, if fewer than 100 cases, the national estimate is used, if more than 500 the state estimate is used, and if between 100 and 500 the distribution is a weighted average of state and national estimates.

The national estimate is obtained via the same method but with no upper limit on the window size and excluding data from Victoria since 14 June, since the situation during the Victorian outbreak after this time is not likely to be representative of surveillance in states with few cases.

#### Macro-distancing model

The population-wide average daily number of non-household contacts at a given time can be directly estimated using a contact survey. We therefore used data from a series of contact surveys commencing immediately after the introduction of distancing restrictions to estimate $\delta_{i}\,\left(t\right)$ independently of case data. To infer a continuous trend of $\delta_{i}\,\left(t\right)$, we model the numbers of non-household contacts at a given time as a function of mobility metrics considered in the mobility model. We model the log of the average number of contacts on each day as a linear model of the log of the ratio on baseline of five Google metrics of time spent at different types of location: residential, transit stations, parks, workplaces, and retail and recreation:

(16) $$\log\left(\delta_{i}\,\left(t\right)\right)=\left(\omega ⊙m\right)\,\log\left(M_{i}\,\left(t\right)\right).$$

where ω is the the vector of 5 coefficients, m is an vector of length 5 containing ones, except for the element corresponding to time at residential locations, which has value -1, and ⊙ indicates the element-wise product. This constrains the direction of the effect of increasing time spent at each of these locations to be positive (more contacts), except for time at residential, which we constrain to be negative. The intercept of the linear model (average daily contacts at baseline) is given an prior formed from the daily number of non-household contacts in a pre-COVID-19 contact survey Rolls et al., 2015. Since our aim is to capture general trends in mobility rather than daily effects, we model the weekly average of the daily number of contacts, by using smoothed estimates of the Google mobility metrics.

Whilst we aim to model weekly rather than daily variation in contact rates, when fitting the model to survey data we account for variation among responses by day of the week by modelling the fraction of the weekly number of contacts falling on each day of the week (the length-seven vector in each state and time $D_{i}\,\left(t\right)$) and using this to adjust the expected number of contacts for each respondent based on the day of the week they completed the survey. To account for how the weekly distribution of contacts has changed over time as a function of mixing restrictions (e.g. a lower proportion of contacts on weekdays during periods when stay-at-home orders were in place), we model the weekly distribution of contacts itself as a function of deviation in the weekly average of the daily number of contacts, with length-seven vector parameters α and θ. We use the softmax (normalised exponential) function to transform this distribution to sum to one, then multiply the resulting proportion by 7 to reweight the weekly average daily contact rate to the relevant day of the week.

Combining the baseline average daily contact rate $N\,C_{0}$, mobility-driven modelled change in contact rates over time $\delta_{i}\,\left(t\right)$, and time-varying day of the week effects $D_{i}\,\left(t\right)$ we obtain an expected number of daily contacts for each survey response $N\,C_{k}$:

(17) $$\log\left(NC_{k}\right)=\log\left(NC_{0}\right)+\log\left(\delta_{i\,\left[k\right]}\left(t\left[k\right]\right)\right)+\log{\left(D_{i\,\left[k\right]}\left(t\left[k\right]\right)*7\right)}_{d\,\left[k\right]}$$

(18) $$D_{i}\,\left(t\right)=\text{softmax}\,\left(\alpha +\theta \,\log\left(\delta_{i}\,\left(t\right)\right)\right)$$

where i⁢[k], t⁢[k], and d⁢[k] respectively indicate the state, time, and day of the week on which respondent k filled in the survey.

We model the number of contacts from each survey respondent as a draw from an interval-censored discrete lognormal distribution. This choice of distribution enables us to account for the ad-hoc rounding of reported numbers of contacts (responses larger than 10 tend to be ‘heaped’ on multiples of 10 and 100), whilst also accounting for heavy upper tail in numbers of reported contacts. The support of this distribution is the integers from 0 to 10 inclusive, and the intervals 11–20, 21–50, and 50–999. Reported daily contact rates ≥ 1000 are excluded as these are considered implausible for our definition of a contact. The probability mass function of this distribution is the integral across these ranges of a lognormal distribution with parameters $\mu_{k}$ and τ, parameterised such that the mean of the distribution is $N\,C_{k}$:

(19) $$\mu_{k}=\log\left(N\,C_{k}\right)-\tau^{2}/2$$

We incorporate mobility data into transmission potential in a two-stage process. In the first stage, non-household contact rates are modelled using mobility and survey data. The posterior mean of the modelled non-household contact rate in each jurisdiction over time is then incorporated in the transmission potential model as a fixed (i.e. ‘data’) timeseries without propagation of posterior uncertainty. Uncertainty in the macro-distancing model could be propagated through to the TP model by estimating both parts in a single joint model. However this would be computationally very burdensome, and long run times would reduce the utility of the transmission potential model for routine situational assessment. Moreover, because uncertainty in both the macro-distancing and transmission potential timeseries are homoscedastic (the posterior variance is more or less constant over time in each state), propagation of the uncertainty in the macro-distancing model is unlikely to have a material effect on estimation of TP timeseries.

#### Precautionary micro-behaviour model

Unlike with macro-distancing behaviour and contact rates, there is no simple mathematical framework linking change in precautionary micro-behaviours to changes in non-household transmission probabilities. We must therefore estimate the effect of precautionary micro-behaviour on transmission via case data. We implicitly assume that any reduction in local-to-local transmission potential that is not explained by changes to the numbers of non-household contacts, the duration of household contacts, or improved disease surveillance is explained by the effect of precautionary micro-behaviour on non-household transmission probabilities.

Whilst it is not necessary to use ancillary data to estimate the effect that precautionary micro-behaviour has at its peak, we use behavioural survey data to estimate the temporal trend in precautionary micro-behaviour, in order to estimate to what extent adoption of that behaviour has waned and how that has affected transmission potential.

We therefore model $\gamma_{t}$ (a time-varying index of change relative to baseline of transmission probability per non-household contact, see Equation (12)), as a function of the proportion of the population adhering to precautionary micro-behaviours. We consider adherence to the ‘1.5 m rule’ as indicative of this broader suite of behaviours due to the availability of data on this behaviour in a series of weekly behavioural surveys beginning prior to the last distancing restriction being implemented Department of the Prime Minister and Cabinet, 2020. We consider the number $m_{i,t}^{+}$ of respondents in region i on survey wave commencing at time t replying that they ‘always’ keep 1.5 m distance from non-household members, as a binomial sample with sample size $m_{i,t}$. We use a generalised additive model to estimate $c_{i}\,\left(t\right)$, the proportion of the population in region i responding that they always comply as a the intervention stage, smoothed over time. Intervention stages are defined as periods of a continuous state of stay-at-home order, and this state thus switches each time a stay-at-home order is started, ended, or significantly changed. This state switching allows the model to react to sudden changes in compliance behaviour when orders are made or rescinded. We assume that the temporal pattern in the initial rate of adoption of the behaviour is the same as for macro-distancing behaviours — the adoption curve estimated from the mobility model. In other words, we assume that all macro-distancing and precautionary micro-behaviours were adopted simultaneously around the time the first population-wide restrictions were put in place in March and April 2020. However we do not assume that these behaviours peaked at the same time or subsequently followed the same temporal trend. The model for the proportion complying with this behaviour is therefore:

(20) $$m_{i,t}^{+}=\mathrm{Binomial}\,\left(m_{i,t},c_{i}\,\left(t\right)\right)$$

(21) $$l\,o\,g\,i\,t\,\left(c_{i}\,\left(t\right)\right)=\zeta_{i,j}+s\,\left(t\right)$$

where $\zeta_{i,j}$ is intervention state j in region i, and s is a smoothing function over time t.

Given $c_{i}\,\left(t\right)$, we model $\gamma_{i}\,\left(t\right)$ as a function of the degree of precautionary micro-behaviour relative to the peak:

(22) $$\gamma_{i}\,\left(t\right)=1-\beta \,\left(c_{i}\,\left(t\right)/\kappa_{i}\right)$$

where $\kappa_{i}$ is the peak of compliance, or maximum of $c_{i}\,\left(t\right)$, and β is inferred from case data in the main $R_{\mathrm{eff}}$ model.

#### Overseas quarantine model

We model the effect of overseas quarantine Q⁢(t) via a monotone decreasing step function with values constrained to the unit interval, and with steps at the known dates $\tau_{1}$ and $\tau_{2}$ of changes in quarantine policy:

(23) $$Q(t)=\left\{ \begin{array}{ll} {}_{1} & t<\tau_{1}\\ q_{2} & \tau_{1}\leq t<\tau_{2}\\ q_{3} & \tau_{2}\leq t \end{array}\right.$$

where $q_{1}>q_{2}>q_{3}$ and all parameters are constrained to the unit interval.

#### Error models

The correlated time-series of deviance between transmission potential and the effective reproduction number for local-to-local transmission in each region $\epsilon_{i}\,\left(t\right)$ is modelled as a zero-mean Gaussian process (GP) with covariance structure reflecting temporal correlation in errors within each region, but independent between regions. We use a Matern 5/2 covariance function k, enabling a mixture of relatively smooth trends and local ’roughness’ to represent the sudden rapid growth of cases that can occur with a high-transmission cluster. Kernel parameters σ and l are the same across regions:

(24) $$\epsilon_{i}∼G\,P\,\left(0,k\,\left(t,t^{\prime }\right)\right)$$

(25) $$k\,\left(t,t^{\prime }\right)=\sigma^{2}\,\left(1+\frac{\sqrt{5}\,\left|t-t^{\prime }\right|}{l}+\frac{5\,{\left(t-t^{\prime }\right)}^{2}}{3\,l^{2}}\right)\,e\,x\,p\,\left(\frac{-\sqrt{5}\,\left|t-t^{\prime }\right|}{l}\right)$$

#### Components of local transmission potential

We model the rate of transmission from locally acquired cases as a combination of the time-varying mechanistic model of transmission rates $R_{i}^{*}\,\left(t\right)$, and a temporally-correlated error term $e^{\epsilon_{i}\,\left(t\right)}$. This structure enables inference of mechanistically interpretable parameters whilst also ensuring that statistical properties of the observed data are represented by the model. Moreover, these two parts of the model can also be interpreted in epidemiological terms as two different components of transmission rates:

1. Component 1 (TP) – transmission rates averaged over the whole state population, representing how macro-distancing, precautionary micro-behaviours, and other factors affect the potential for widespread community transmission ($R_{i}^{*}\,\left(t\right)$), and
2. Component 2 (C2) – the degree to which the transmission rates of the population of current active cases deviates from the average statewide transmission rate ($e^{\epsilon_{i}\,\left(t\right)}$).

Component 2 reflects the fact that the population of current active cases in each state at a given time will not be representative of the the state-wide population, and may be either higher (e.g. when cases arise from a cluster in a high-transmission environment) or lower (e.g. when clusters are brought under control and cases placed in isolation).

Component 1 (TP) can therefore be interpreted as the expected rate of transmission if cases were widespread (population-representative) in the community. The product of Components 1 and 2 ($R_{\mathrm{eff}}$) can be interpreted as the rate of transmission in the sub-population making up active cases at a given time.

Where a state has active cases in one or more clusters, the combination of these components gives the apparent rate of transmission in those clusters ($R_{\mathrm{eff}}$), given by Equation 10. This reflects the interpretation that TP captures the population mean of a distribution over individual-level reproduction numbers, and $R_{\mathrm{eff}}$ is the mean of a (non-random) sample from that distribution — the population comprising cases at that point in time. While not used in the public health context in Australia, the epidemiological interpretation of the $R_{\mathrm{eff}}$ when a state has no active cases is the rate of spread expected if an index case were to occur in a random sub-population. Because the amplitude of this error term is learned from the data, this is informative as to the range of plausible rates of spread that might be expected from a case being introduced into a random sub-population. However, the mean of this distribution, TP, may play a similar role and has proven to be a more interpretable quantity for end users of this model.

#### Parameter values and prior distributions

The parameters of the generation interval distribution are the posterior mean parameter estimates corresponding to a lognormal distribution over the serial interval estimated by Nishiura et al., 2020. The shape of the generation interval distribution for SARS-CoV-2 in comparable populations is not well understood, and a number of alternative distributions have been suggested by other analyses. A sensitivity analysis performed by running the model with alternative generation interval distributions (not presented here) showed that parameter estimates were fairly consistent between these scenarios, and the main findings were unaffected. A full, formal analysis of sensitivity to this and other assumptions will be presented in a future publication.

No ancillary data are available to inform p, the probability of transmission per hour of contact in the absence of distancing behaviour. However, at t=0, holding $H\,C_{0}$, $N\,C_{0}H\,D_{0}$, and $N\,D_{0}$ constant, there is a deterministic relationship between p and $R_{i}^{*}\,\left(0\right)$ (the basic reproduction number, which is the same for all states). The parameter p is therefore identifiable from transmission rates at the beginning of the first epidemic wave in Australia. We define a prior on p that corresponds to a prior over $R_{i}^{*}\,\left(0\right)$ matching the averages of the posterior means and 95% credible intervals for 11 European countries as estimated by Flaxman et al., 2020 in a sensitivity analysis where the mean generation interval was 5 days — similar to the serial interval distribution assumed here. This corresponds to a prior mean of 2.79, and a standard deviation of 1.70 for $R_{i}^{*}\,\left(0\right)$. This prior distribution over p was determined by a Monte-Carlo moment-matching algorithm, integrating over the prior values for $H\,C_{0}$, $N\,C_{0}H\,D_{0}$, and $N\,D_{0}$.

#### Model fitting

We fitted (separate) models of $c_{i}\,\left(t\right)$ and $N\,C_{0}\,\delta_{i}\,\left(t\right)$ to survey data alone in order to infer trends in those parameters as informed by survey data. These are shown in Figure 3. We used the posterior means of each of these model outputs as inputs into the $R_{\mathrm{eff}}$ model. The posterior variance of each of these quantities is largely consistent over time and between states, and the absolute effect of each is scaled by other parameters (e.g. β), meaning that uncertainty in these quantities is largely not identifiable from uncertainty in other scaling parameters. As a consequence, propagation of uncertainty in these parameters into the $R_{\mathrm{eff}}$ model (as was performed in a previous iteration of the model) has little impact on estimates of $R_{\mathrm{eff}}$ and transmission potential, so is avoided for computational brevity.

Inference was performed by Hamiltonian Monte Carlo using the R packages greta and greta.gp 5 (Golding, 2019; Golding, 2020). Posterior samples of model parameters were generated by 10 independent chains of a Hamiltonian Monte Carlo sampler, each run for 1000 iterations after an initial, discarded, ‘warm-up’ period (1000 iterations per chain) during which the sampler step size and diagonal mass matrix was tuned, and the regions of highest density located. Convergence was assessed by visual assessment of chains, ensuring that the potential scale reduction factor for all parameters had values less than 1.1, and that there were at least 1000 effective samples for each parameter.

Visual posterior predictive checks were performed to ensure that the observed data were consistent with the posterior predictive density over all cases (and survey results), and over time-varying case predictions within each state.

### Code availability

Model code for performing the analyses and generating the figures is available at: https://github.com/goldingn/covid19_australia_interventions, (copy archived at swh:1:rev:9fe78353a2ee6ab9c3b9ed35c1feea6935af769a; Golding, 2023).

## Data Availability

Datasets analysed and generated during this study are available at the following link: https://doi.org/10.26188/19517986.v1. For estimates of the time-varying effective reproduction number and transmission potential (Figure 2), the complete line listed data within the Australian national COVID-19 database are not publicly available. However, we provide the cases per day by notification date and state (Data files 1 and 2) which, when supplemented with the estimated distribution of the delay from symptom onset to notification as in Figure 3D and H (provided in Data files 3 and 4), and Data files 5-10, analyses of the time-varying effective reproduction number and transmission potential can be performed. Data files 5-10 contain the numerical data, output from each of the model components, used to generate Figure 3. For access to the raw data, a request must be submitted via NNDSS.datarequests@health.gov.au which will be assessed by a data committee. Model code for performing the analyses and generating the figures is available at: https://github.com/goldingn/covid19_australia_interventions (copy archived at swh:1:rev:9fe78353a2ee6ab9c3b9ed35c1feea6935af769a). The following dataset was generated: GoldingN PriceDJ RyanG McVernonJ McCawJM ShearerFM 2023Data files to support manuscript: A modelling approach to estimate the transmissibility of SARS-CoV-2 during periods of high, low and zero case incidencefigshare10.26188/19517986.v1PMC999511236661303

## Additional files

MDAR checklist

Supplementary file 1. Dates of key changes in restrictions on gatherings and movement for New South Wales and Victoria, as shown in Figures 2 and 3.

Supplementary file 2. Parameters in the semi-mechanistic part of the time-varying model of $R_{eff}$. Prior on weights for ω correspond to Google mobility metrics in the following order: parks, residential, retail and recreation, transit stations, workplaces.

Supplementary file 3. Fixed parameters in the semi-mechanistic part of the time-varying model of $R_{eff}$.

Supplementary file 4. Parameters used in the time-series part of the time-varying model of $R_{eff}$.
