# Supplementary material for: A modelling approach to estimate the transmissibility of SARS-CoV-2 during periods of high, low, and zero case incidence
Source: eLife. 2023 Jan 20;12:e78089. doi: 10.7554/eLife.78089 (PMC9995112; doi:10.7554/eLife.78089)
Supplement: Peer review history

# Editor report: Editor's evaluation

**DOI:** 10.7554/eLife.78089.sa0

**Authors:** Caroline Colijn

**Affiliations:** https://ror.org/0213rcc28 Simon Fraser University Canada

This paper is interesting, timely, and important because it presents a way to understand the transmission potential of a virus even when there are very few local cases. This has a high public health communication and preparedness value. The paper is clearly written, and the results fit with the known epidemiology of outbreaks that occurred in Australia in 2020. The results are convincing and likely to be of broad interest within and outside the field of epidemiological modelling.

# Decision letter

**DOI:** 10.7554/eLife.78089.sa1

**Affiliations:** https://ror.org/0213rcc28 Simon Fraser University Canada; https://ror.org/03y7q9t39 University of Canterbury New Zealand; https://ror.org/04haebc03 Memorial University of Newfoundland Canada

Our editorial process produces two outputs: (i) public reviews designed to be posted alongside the preprint for the benefit of readers; (ii) feedback on the manuscript for the authors, including requests for revisions, shown below. We also include an acceptance summary that explains what the editors found interesting or important about the work.

**Decision letter after peer review:**

Thank you for submitting your article "A modelling approach to estimate the transmissibility of SARS-CoV-2 during periods of high, low, and zero case incidence" for consideration by *eLife*. Your article has been reviewed by 2 peer reviewers, and the evaluation has been overseen by a Reviewing Editor and a Senior Editor. The following individuals involved in review of your submission have agreed to reveal their identity: Michael Plank (Reviewer #1); Amy Hurford (Reviewer #2).

As is customary in *eLife*, the reviewers have discussed their critiques with one another. What follows below is the Reviewing Editor's edited compilation of the essential and ancillary points provided by reviewers in their critiques and in their interaction post-review. Please submit a revised version that addresses these concerns directly. Although we expect that you will address these comments in your response letter, we also need to see the corresponding revision clearly marked in the text of the manuscript. Some of the reviewers' comments may seem to be simple queries or challenges that do not prompt revisions to the text. Please keep in mind, however, that readers may have the same perspective as the reviewers. Therefore, it is essential that you attempt to amend or expand the text to clarify the narrative accordingly.

Essential revisions:

We all agree that this work is of interest and is likely to be suitable for publication in *eLife*.

1) Most of the reviewer questions are clarifications. Could you please address these more detailed comments?

2) We had some discussion about the terminology re: micro-distancing, whether it's really about *distancing*, mask use, ventilation etc. In particular, one reviewer noted that the focus on 1.5m distancing should be better justified given what we know about airborne transmission – presumably, this survey question should be interpreted as a proxy for precautionary micro-behaviour (which may include mask use, preference for outdoor or well ventilated locations,) rather than a mechanistic metric for transmission risk which we know is not primarily determined by distance.

Would you consider potential alternative terminology to make it clear it's about more than just distance per se? E.g. some modelling groups use the term "precautionary behaviour" for a similar inferred quantity.

We are aware, though, that these terms may be established in Australia, and we don't wish to introduce more confusion.

(3) A clear statement of the minimal data requirements or implications of implementing the framework with reduced data availability would be a helpful addition.

*Reviewer #1 (Recommendations for the authors):*

For the VIC second wave, the observation that Reff>TP is explained as being due to the nature of the sub-population the virus was predominantly spread in. That's certainly plausible epidemiologically. However another interpretation is presumably that the TP model is systematically underestimating TP. Can this be ruled out based on the result of the model?

The model description in the supplementary left me unclear about how mobility data and survey data were combined to estimate macro-distancing behaviour. This part of the model could do with some clarification. E.g. Were these used simultaneously or sequentially? From Equation 16 and line 630 it appears there is a deterministic relation between mobility data M(t) and number of non-household contacts δ(t). Were the survey data used in the process of estimating the coefficients m? Presumably the mapping between survey data and mobility data is noisy – is there an implicit noise term or residual in equation 16? Line 433 says waning in macro-distancing is driven by mobility data – so how does survey data come into estimating this? In Figure S5, it appears that the posterior estimates for macro-distancing for NSW and QLD are systematically higher than the survey data – is this because the mobility data is pulling these estimates back up, i.e. mobility data in these states tend to be closer to baseline, for the same number of reported mean non-household contacts?

Similarly regarding the micro-distancing model – Line 448 – "infer the date of peak micro-distancing behaviour" and the rate of waning. Does this mean micro-distancing is assumed to follow some parametric functional form with respect to time? Or otherwise constrained to have a single peak followed by a waning phase? How does this relate to Equation 21, where micro-distancing appears to be a function solely of the "intervention state" in that region. So wouldn't the timing of interventions in different regions determine when the peak micro-distancing occurred? And how does/would this assumption work if the survey data showed that in fact actual behaviour was not highly correlated with interventions (which it may have been in March 2020 but could conceivably become less so over time). How were the xi_ij parameters in (21) estimated (there doesn't seem to be a prior specified for them)? The micro-distancing behaviour is described as mainly relating to observation of the "1.5m distancing rule". Another significant behaviour factor affecting probability of transmission during a non-household contact is mask use yet this is not mentioned. Is there a reason this wasn't considered in the model, e.g. the survey did not ask about masks? or is it that the effect of masks could not be separately estimated?

Other specific comments

– Is transmission/ travel between different states accounted for or ignored?

– The references to the panels of Figure 2 seem to have got mixed up as the text consistently refers to panels B and F as TP and C and G as Reff but the Figure has them the other way round.

– Line 174 "Reff dropped below 1… prior to activation of stay-at-home restrictions". The data at which Reff dropped below 1 presumably could be given a confidence interval based on the green bands in Figure 2. Do these CIs overlap the date of introduction of stay-at-home restrictions? I also wonder how sensitive these inferred dates are to the infection to reporting lag and the degree of smoothness that is a priori imposed on Reff(t).

– Figure 3 caption mentions the blue bar but this seems to be missing from the graphs. Also it is a bit confusing that both the macro-distancing graphs (B and F) and micro-distancing (C and G) are described "reduction in…" when distancing behaviour appears to be associated with a decrease in B and F but an increase in C and G.

– There appears to be some notational inconsistency or at least ambiguity in the supplementary section. E.g. is TP synonymous with R*(t) in Equation 10? Is Ri^L(t) (or some combination of R^L and R^O) in Equation 6 the same as Reff(t)? Clarifying this would help understand how the method actually estimated these quantities from the data.

– In Equation 10 sigma2 appears to have the effect of always reducing RL (effective reproduction number?) relative to R* (TP). Or do the epsilons have strictly positive mean? In the absence of random effects (epsilon=0) wouldn't you expect R* and RL to have the same mean?

– In Equation (12) is surveillance assumed to have the same effect on household and non-household transmission? If so is that realistic given it's hard to isolate from people at home?

– In Equation (14) f is described as a probability density but I think it must actually be a survival function (or 1- CDF) i.e. f(t') = P(case not yet isolated at time t' after infection). Otherwise Equation (14) can't be correct, e.g. if all cases were isolated on day t'=5 that would say g(t')=0 for t'!=5 rather than g(t')=g*(t') for t'<5 and 0 for t'>5.

– Line 636 should the element corresponding to residential have value -1 not 1 so it is opposite sign to the non-residential locations? And are the 5 coefficients in w constrained to be non-negative?

– Paragraph following line 681 – is there a reason only the "always" response was used?

– Line 748 – is estimating p from transmission rates at the beginning if 1st wave representative of subsequent times? Given the concentration in overseas arrivals one might expect this to be different? Or is it because it's per hour of contact so number of contacts are factored out?

– Figure S8 – it appears there is no change in effect at the second intervention why is this? And the black dotted line mentioned in the caption does not seem to be there.

*Reviewer #2 (Recommendations for the authors):*

This is valuable work that fills an important need – thank you for doing this!

Future work may consider how to make this approach more accessible by outlining minimum data needs, data collection priorities, or describing the implications of proceeding with the transmission potential calculation even if a data source is unavailable (i.e. for estimation of micro-distancing). Your approach is rigorous, but also more similar to a complete model of infection spread, rather than a quick calculation of a summary statistic during a real-time pandemic response in a region with few modellers and limited resources (i.e. the Pacific Islands, or Atlantic Canada and Canada's northern territories, with much fewer resources than Australia, but that still had an important need for this approach during the pandemic).

In Table 1, regarding the heading "Local elimination", perhaps "Local transmission" is more appropriate since fundamentally this column discusses local transmission, and elimination is non-essential.

# Reply: Author response

**DOI:** 10.7554/eLife.78089.sa2

Essential revisions:

We all agree that this work is of interest and is likely to be suitable for publication in eLife.

1) Most of the reviewer questions are clarifications. Could you please address these more detailed comments?

We respond to each of these clarifications separately below.

2) We had some discussion about the terminology re: micro-distancing, whether it's really about distancing, mask use, ventilation etc. In particular, one reviewer noted that the focus on 1.5m distancing should be better justified given what we know about airborne transmission – presumably, this survey question should be interpreted as a proxy for precautionary micro-behaviour (which may include mask use, preference for outdoor or well ventilated locations,) rather than a mechanistic metric for transmission risk which we know is not primarily determined by distance.

Would you consider potential alternative terminology to make it clear it's about more than just distance per se? E.g. some modelling groups use the term "precautionary behaviour" for a similar inferred quantity.

We are aware, though, that these terms may be established in Australia, and we don't wish to introduce more confusion.

We agree with these important points. The modelling framework uses adherence to the 1.5m rule as a proxy for all behaviours (other than reducing the number of contacts, test-seeking etc.) that may influence transmission, and so is intended to capture the use of masks and preference for outdoor meetings. Adherence to the 1.5m rule was a convenient metric of these risk-avoidance behaviours as this has been consistent public health advice since the beginning of the pandemic in Australia, enabling us to track this metric over time for the entire duration. Comparison between adherence to the 1.5m rule and mask-wearing has indicated that they tend to follow the same pattern; ie. increasing in response to lockdown-type restrictions and spikes in case counts. We have added the following wording to the manuscript to clarify this point (line 100-108):

The modelling framework uses adherence to the 1.5 metre rule as a proxy for all behaviours (other than those reducing the number of contacts) that may influence transmission, and so is intended to capture the use of masks, preference for outdoor gatherings, and hand hygiene, among other factors. The 1.5 metre rule was a suitable proxy because it was consistent public health advice throughout the analysis period and time-series data were available to track adherence to this metric over time.

Furthermore, we have adjusted our terminology to “precautionary micro-behaviour” throughout the manuscript to improve clarity. However, we note on lines (X-X) that the term “micro-distancing” has been used for Australian reporting purposes.

(3) A clear statement of the minimal data requirements or implications of implementing the framework with reduced data availability would be a helpful addition.

The framework we describe here was iteratively developed throughout the pandemic in Australia, in order to synthesise available data relating to the transmission process and to address situation-specific questions. The framework is therefore inherently modular, and could be modified to incorporate or remove time-series of relevant quantities (e.g. non-household contact rates, adherence to precautionary micro-behaviour, effectiveness of surveillance), as available. For its use in Australia in 2020, non-household contact rates (capturing the main effects of lockdown-type measures) and precautionary micro-behaviour were likely the most important aspects. However in other times and places with different drivers of epidemic dynamics, other factors may be more important. The variables that are important to quantify and include should therefore be chosen accordingly. We have added wording to the discussion to make these points (lines 349-359):

“The requirement for specific data streams is a limitation of our approach routinely applied in Australia in 2020 --- where it was developed to address situation-specific policy questions and synthesise available data relating to the transmission process. However, the framework is modular and could be adjusted to incorporate or remove time-series of relevant quantities (e.g., non-household contact rates, adherence to precautionary micro-behaviour, effectiveness of surveillance), according to data availability, epidemiological relevance, and policy needs. For its use in Australia in 2020, non-household contact rates (capturing the main effects of stay-at-home measures) and precautionary micro-behaviour were considered the most important (and measurable) drivers of epidemic dynamics. In other times and places (or for other diseases), different factors may be more important for monitoring epidemic dynamics, and the variables that are quantified should be chosen accordingly.”

Reviewer #1 (Recommendations for the authors):

For the VIC second wave, the observation that Reff>TP is explained as being due to the nature of the sub-population the virus was predominantly spread in. That's certainly plausible epidemiologically. However another interpretation is presumably that the TP model is systematically underestimating TP. Can this be ruled out based on the result of the model?

This is an important point. The model is designed such that on average over the long term, Reff is approximately equal to TP. However this does not preclude that modelled TP could be systematically underestimating the ‘true’ TP. However since TP is a theoretical, rather than a directly measurable quantity, it was not possible to quantitatively validate this part of the model in the epidemiological situation in Australia in 2020, since there was no widespread transmission in any jurisdiction. We have added to existing text in the Discussion to clarify this (line 369-372).

Existing text: “While the patterns of TP, Reff and C2 observed over time in Australia are consistent with “in field'' epidemiological assessments, and while the methods have demonstrated impact in supporting decision making, a direct quantification of the validity of the TP is not straightforward. For example, whether self-reported adherence to the 1.5 m rule is a reliable covariate for change in the per contact probability of transmission over time is difficult to assess. If transmission were to become widespread in Australia; and therefore cases become more representative of the general population rather than specific subsets, Reff and TP estimates would be expected to converge. However in the absence of such a natural experiment, no ground truth for this unobserved parameter exists with which to quantitatively validate the model calibration.”

New text: “During the Victorian second wave, while Reff > TP is consistent with virus spread in sub-populations with higher-than-population-average rates of social contact, which was supported by other epidemiological assessments, we cannot rule out that the modelled TP was systematically underestimating the `true' TP over this period.”

The model description in the supplementary left me unclear about how mobility data and survey data were combined to estimate macro-distancing behaviour. This part of the model could do with some clarification. E.g. Were these used simultaneously or sequentially? From Equation 16 and line 630 it appears there is a deterministic relation between mobility data M(t) and number of non-household contacts δ(t). Were the survey data used in the process of estimating the coefficients m? Presumably the mapping between survey data and mobility data is noisy – is there an implicit noise term or residual in equation 16?

Thank you for highlighting this unclear wording. We have edited the text to clarify that the models are used sequentially: non-household contact rates are modeled first and then the prediction used in the TP/Reff model; and that uncertainty in the non-household contact rates are not explicitly propagated into the TP/Reff model, because this uncertainty is absorbed by other parameters in the TP/Reff model. We initially fitted these models simultaneously, enabling full propagation of uncertainty, however this model-fitting became computationally infeasible as the time-series grew and provided no material benefit. We believe this information would be of interest to other modellers, and so have described it in more detail on lines 725-735 as follows:

We incorporate mobility data into transmission potential in a two-stage process. In the first stage, non-household contact rates are modeled using mobility and survey data. The posterior mean of the modeled non-household contact rate in each jurisdiction over time is then incorporated in the transmission potential model as a fixed (i.e. ‘data’) timeseries without propagation of posterior uncertainty. Uncertainty in the macro-distancing model could be propagated through to the TP model by estimating both parts in a single joint model. However this would be computationally very burdensome, and long run times would reduce the utility of the transmission potential model for routine situational assessment. Moreover, because uncertainty in both the macro-distancing and transmission potential timeseries are homoscedastic (the posterior variance is more or less constant over time in each state), propagation of the uncertainty in the macro-distancing model is unlikely to have a material effect on estimation of TP timeseries.

Line 433 says waning in macro-distancing is driven by mobility data – so how does survey data come into estimating this? In Figure S5, it appears that the posterior estimates for macro-distancing for NSW and QLD are systematically higher than the survey data – is this because the mobility data is pulling these estimates back up, i.e. mobility data in these states tend to be closer to baseline, for the same number of reported mean non-household contacts?

We have edited the explanation of the macro-distancing model to clarify how it is fitted to the survey data. We also note that the bars indicating pointwise estimates of non-household contact rates are not in fact the raw data, but the output of a different model. The reason for this is that empirical averages (or other non-model-based summaries) of non-household contact numbers in surveys are highly skewed and volatile: while most respondents have very few contacts, occasionally respondents report hundreds of contacts. Since these responses occur in some weeks and not others, week-by-week estimates can fluctuate wildly. Model fitting employs a specific likelihood model to account for these data, but this makes visual comparison of data and model fit very difficult. The pointwise estimates are included to indicate data sparsity and uncertainty (being larger in jurisdictions with fewer samples), rather than fit to data. The new wording is as follows (lines 462–464):

“Waning in macro-distancing behaviour is therefore driven by Google mobility data (calibrated to survey data on non-household contact rates) on increasing time spent in each of the different types of locations since the peak of macro-distancing behaviour.”

The following has been added to the Figure S5 legend:

“The pointwise estimates for each survey round represented as black lines and grey rectangles are the outputs of a separate statistical model that does not include the mobility data covariates, and is intended as a visual illustration of the level of data sparsity and variability, rather than a way of estimating fit to data, since the raw data for each week is subject to significant skew.”

Similarly regarding the micro-distancing model – Line 448 – "infer the date of peak micro-distancing behaviour" and the rate of waning. Does this mean micro-distancing is assumed to follow some parametric functional form with respect to time? Or otherwise constrained to have a single peak followed by a waning phase? How does this relate to Equation 21, where micro-distancing appears to be a function solely of the "intervention state" in that region. So wouldn't the timing of interventions in different regions determine when the peak micro-distancing occurred? And how does/would this assumption work if the survey data showed that in fact actual behaviour was not highly correlated with interventions (which it may have been in March 2020 but could conceivably become less so over time). How were the xi_ij parameters in (21) estimated (there doesn't seem to be a prior specified for them)? The micro-distancing behaviour is described as mainly relating to observation of the "1.5m distancing rule".

Apologies for this confusing text, this was accidentally copied over from a report regarding an earlier iteration of this model that used an alternative model with the aim of predicting the peak of microdistancing. For the version of the TP model framework presented here, we used Generalised Additive Models to model micro-distancing behaviour, as detailed in the Methods section. We have deleted the confusing final sentence of this paragraph.

Another significant behaviour factor affecting probability of transmission during a non-household contact is mask use yet this is not mentioned. Is there a reason this wasn't considered in the model, e.g. the survey did not ask about masks? or is it that the effect of masks could not be separately estimated?

As detailed and clarified above, we included the 1.5m rule as a metric of precautionary micro-behaviour because data on adherence was available for the duration of the pandemic, and it is likely to be correlated with other similar behaviours like mask wearing. Because mask-wearing was not initially a part of the official health advice in Australia, data on adherence was not collected in the early stages of the pandemic. This dataset is therefore not ideally suited to distinguishing the marginal effect on transmission of mask-wearing, from the effect of the broader suite of precautionary micro-behaviours.

Other specific comments

– Is transmission/ travel between different states accounted for or ignored?

Each jurisdiction is modelled as a separate epidemic. Travel is only considered between states when accounting for the place of acquisition of cases. Cases acquired interstate are considered as “imported cases” within the modelling framework, that is, they do not arise from locally acquired cases but can contribute to onward local transmission. A description of how imported versus locally-acquired cases are handled within the modelling framework is provided in equations 1-8 in the Methods section. Given Australia’s unique geography (the majority of Australians live in a handful of major cities, with comparatively little movement between them), and the implementation of interstate travel restrictions during periods of transmission, the number of interstate importations in Australia was small, and well documented in the data. This may not be the case in other settings.

We have added the following to the text to make this clearer under a new sub-heading

“Accounting for the impact of interstate-acquired infections” (lines 501-510):

“Each of Australia's eight states and territories were modelled as a separate epidemic, with no travel assumed between jurisdictions and interstate-acquired cases handled as “imported cases" within the modelling framework (but contributing to the case counts in their jurisdiction of origin for the model likelihood). We believe that these modelling decisions were reasonable for the Australian context given Australia's unique geography (the majority of Australians live in a handful of major cities, with comparatively little movement between them), and the imposition of interstate travel restrictions during periods of COVID-19 transmission over the analysis period. Furthermore, the number of interstate importations in Australia was small and well documented in the data. Unlike overseas-acquired cases, interstate-acquired cases are assumed to contribute to onward local transmission since they were not required to quarantine.”

– The references to the panels of Figure 2 seem to have got mixed up as the text consistently refers to panels B and F as TP and C and G as Reff but the Figure has them the other way round.

Thank you. We have corrected the text referring to Figure 2.

– Line 174 "Reff dropped below 1… prior to activation of stay-at-home restrictions". The data at which Reff dropped below 1 presumably could be given a confidence interval based on the green bands in Figure 2. Do these CIs overlap the date of introduction of stay-at-home restrictions? I also wonder how sensitive these inferred dates are to the infection to reporting lag and the degree of smoothness that is a priori imposed on Reff(t).

This is an important point. However, the confidence intervals do not overlap with the date of introduction of stay-at-home restrictions. Our estimates of Reff account for the lag from infection to reporting and any impact of statistical smoothing would have shifted this segment of the Reff time-series to the right i.e. closer to the date of imposition of stay-at-home restrictions. We have edited the sentence to improve clarity, now reporting the date on which the upper confidence interval crossed 1 (instead of the median) and reporting the date that the stay-at-home restrictions were imposed (8 days later) (line 181-183):

“Our method, with its ability to distinguish between import-to-local and local-to-local transmission, estimates that the local Reff dropped below 1 on 22 March (upper confidence intervals) in both Victoria and New South Wales --- prior to the activation of stay-at-home restrictions on 30 March.”

– Figure 3 caption mentions the blue bar but this seems to be missing from the graphs. Also it is a bit confusing that both the macro-distancing graphs (B and F) and micro-distancing (C and G) are described "reduction in…" when distancing behaviour appears to be associated with a decrease in B and F but an increase in C and G.

Thank you for noting that the blue bar is missing from Figure 3. We have now corrected this. To further aid in interpretation of Figures 2 and 3, we have also added a column to Table S1, “Label”, where we include the letter associated with each vertical line in Figures 2 and 3.

– There appears to be some notational inconsistency or at least ambiguity in the supplementary section. E.g. is TP synonymous with R*(t) in Equation 10? Is Ri^L(t) (or some combination of R^L and R^O) in Equation 6 the same as Reff(t)? Clarifying this would help understand how the method actually estimated these quantities from the data.

That is correct. R(t) denotes Reff, with R^L(t) and R^O(t) respectively the Reffs of locally-acquired and overseas acquired infections. R*(t) is transmission potential. We have edited the text immediately below Equation 10 to make this clearer:

“For both locally-acquired and overseas-acquired infections, the effective reproduction number depends on the transmission potential R_i*(t). R_i*(t) is given by a deterministic epidemiological model of population-wide transmission potential that considers the effects of distancing behaviours.”

– In Equation 10 sigma2 appears to have the effect of always reducing RL (effective reproduction number?) relative to R* (TP). Or do the epsilons have strictly positive mean? In the absence of random effects (epsilon=0) wouldn't you expect R* and RL to have the same mean?

In reviewing this we spotted a typo in this equation. The correct equation should have the σ^2 term divided by 2, and this has been corrected. The correct version of the equation produces R^L that is drawn from a lognormal distribution with mean R*. We provide the working here to clarify our following response, but omit this working in the manuscript, for brevity:

R^L is lognormally-distributed with parameters mu and σ: log(R^L) ~ N(mu, σ^2)

which is equivalent in distribution (via affine transformation of a standard normal) to:

R^L = exp(mu + epsilon) where epsilon has variance σ^2 (as stated in the manuscript) epsilon ~ N(0, σ^2)

The mean of a lognormal distribution is given as:

mean(R^L) = exp(mu + σ^2 / 2)

So setting the mean to R* and solving for mu, we obtain:

mu = log(R*) – σ^2 / 2 And the full equation 10 is:

R^L = exp(log(R*) – σ^2 / 2 + epsilon)

This means that a priori we expect R^L to have marginal long-term mean R*. Ie. if we were to simulating from the priors, conditional on R*, the timeseries trends for R^L (Reff) would go up and down at random, but on average over time would have lognormal distribution, and have long-term mean R*. Therefore it does not follow that we would expect R^L = R* in the absence of the random variates (epsilon = 0), since the lognormal distribution is asymmetric. If epsilon = 0, then R^L would be at the median of the lognormal distribution we intend, which for the lognormal distribution is always lower than the mean.

We believe the text below this equation is accurate in detailing this relationship, but we recognise that the interpretation of this temporal random effect is different from most uses of temporal random effects in statistical modelling; where the effect mops up ‘error’, but does not have a mechanistic interpretation in terms of the distribution of a sample. We have therefore added the following text on lines (588-594) to make this point:

“Note that in this model the random effects term $\epsilon_i$ and its variance term $\σ^2$ is intended to have a mechanistic interpretation as the stochasticity due to random sampling (of people currently infected from the total population). It is not incorporated to account for error in specification of the transmission potential in the way that temporal random effects are commonly used in statistical modelling. Consequently, small variance in the timeseries plots of $\epsilon_i$ is not indicative of good fit, but of a large number of infections; as the size of the sample increases, the variance of mean decreases.”

– In Equation (12) is surveillance assumed to have the same effect on household and non-household transmission? If so is that realistic given it's hard to isolate from people at home?

Yes, that is correct. Throughout the pandemic, government isolation advice in Australia explicitly outlined the need to isolate from others within the same household. However it is possible that isolation reduced within-household transmission less than between-household transmission. We are not aware of any detailed longitudinal household data for Australia that could be used to estimate the effectiveness of isolation within households in order to confirm or quantify this.

– In Equation (14) f is described as a probability density but I think it must actually be a survival function (or 1- CDF) i.e. f(t') = P(case not yet isolated at time t' after infection). Otherwise Equation (14) can't be correct, e.g. if all cases were isolated on day t'=5 that would say g(t')=0 for t'!=5 rather than g(t')=g*(t') for t'<5 and 0 for t'>5.

You are correct, it should say that this is the survival function of the distribution. The wording has been updated to make this clear on line 648:

“We model both of these functions using a region- and time-varying estimate of the survival function (one minus the cumulative density function) f_i(t, t’) of the discrete probability distribution over times from infection to detection: “

– Line 636 should the element corresponding to residential have value -1 not 1 so it is opposite sign to the non-residential locations? And are the 5 coefficients in w constrained to be non-negative?

Yes, that is correct. The minus sign was lost somewhere in formatting. This has now been corrected.

– Paragraph following line 681 – is there a reason only the "always" response was used?

This choice was largely arbitrary, since the aim was to capture temporal patterns in broader precautionary micro-behaviour, rather than an absolute value. Temporal patterns for the proportion giving the ‘always’ response were very similar to those using other thresholds.

– Line 748 – is estimating p from transmission rates at the beginning if 1st wave representative of subsequent times? Given the concentration in overseas arrivals one might expect this to be different? Or is it because it's per hour of contact so number of contacts are factored out?

By separately modelling the rates of transmission from overseas arrivals and local transmission, and by accounting for differences in contact rates and durations, and other behaviours, the parameter p can be interpreted as a virus (and variant) specific parameter.

– Figure S8 – it appears there is no change in effect at the second intervention why is this? And the black dotted line mentioned in the caption does not seem to be there.

The model has the ability to estimate the differences between these periods, with exponential priors that imply a priori that small changes are more likely than big ones. The fact that the posterior for the difference at the second interventions is essentially zero, probably reflects the inference procedure selecting a more parsimonious parameter estimate. We cannot think of a particular epidemiological reason to support this finding. This part of the model is intended to account for changing importation effects in the early part of the pandemic in Australia rather than when estimating local transmission rates. These parameters are of lesser interest to policymakers and are fitted to limited data. We would therefore advise not interpreting these particular parameters too closely. We have added the following wording to express this on lines 497-500:

“Note that this part of the model is intended to capture broad changes in the contribution of importation to case numbers, and is not intended to provide reliable inferences about the relative contributions of different border quarantine policies to disease importation.”

We also have deleted the sentence about the dotted line, which is not visible given the low y-axis.

Reviewer #2 (Recommendations for the authors):

This is valuable work that fills an important need – thank you for doing this!

Thank you for the positive comments.

Future work may consider how to make this approach more accessible by outlining minimum data needs, data collection priorities, or describing the implications of proceeding with the transmission potential calculation even if a data source is unavailable (i.e. for estimation of micro-distancing). Your approach is rigorous, but also more similar to a complete model of infection spread, rather than a quick calculation of a summary statistic during a real-time pandemic response in a region with few modellers and limited resources (i.e. the Pacific Islands, or Atlantic Canada and Canada's northern territories, with much fewer resources than Australia, but that still had an important need for this approach during the pandemic).

We agree. A potential future extension would be modular software that would enable users to input different data sources (not all might be necessary) and lower the technical hurdles to implementation. We have added text noting this to the discussion (lines 398-404):

“These various additions and the component models of our framework (Figure 1) provide a suite of interoperable modules that could be used to apply the transmission potential modelling framework to future epidemic diseases and other settings. Enabling the broader application and uptake of these methods would be aided by the development of robust research software, with the ability to modify which modules are used, to match the data streams available to the analyst. The development of such software, and detailed description of data inputs and analysis of the value of each datastream will be the focus of future work.”

In Table 1, regarding the heading "Local elimination", perhaps "Local transmission" is more appropriate since fundamentally this column discusses local transmission, and elimination is non-essential.

This column is intended to highlight the interpretation of each metric in situations where there is community transmission (denoted “Community transmission”) versus no transmission (denoted “Local elimination”). Since the far right column is describing interpretation for situations where there is no local/community transmission, “local transmission” is not an appropriate substitute for “local elimination”. However to improve clarity, we have adjusted “local elimination” to “no transmission”.
